# Tracing the origin of near-infrared emissions emanating from manganese (II)

**DOI:** 10.1038/s41377-025-01816-y

**Published:** 2025-05-13

**Authors:** Yu Xiao, Xun Yang, Hao-Ran Zhao, Dan Wu, Ming-Xing Chen, Tianxiang Zheng, Rui Zhang, Ling-Dong Sun, Chun-Hua Yan

**Affiliations:** 1 Beijing National Laboratory for Molecular Sciences, State Key Laboratory of Rare Earth Materials Chemistry and Applications, PKU-HKU Joint Laboratory in Rare Earth Materials and Bioinorganic Chemistry, College of Chemistry and Molecular Engineering, Peking University, Beijing, 100871 China; 2 College of Science, Nanjing Forestry University, Nanjing, 210037 China; 3 College of Materials Science and Engineering, Nanjing Forestry University, Nanjing, 210037 China; 4 School of Physical Science and Technology, Inner Mongolia Key Lab of Nanoscience and Nanotechnology, Inner Mongolia University, Hohhot, 010021 China; 5 Analytical Instrumentation Center of Peking University, Beijing, 100871 China; 6 School of Chemical and Environmental Engineering, Shanghai Institute of Technology, Shanghai, 201418 China

**Keywords:** Near-infrared spectroscopy, Optical materials and structures

## Abstract

The enduring enigma surrounding the near-infrared (NIR) emission of Mn^2+^ continues to ignite intense academic discussions. Numerous hypotheses have emerged from extensive research endeavors to explain this phenomenon, such as the formation of Mn^2+^–Mn^2+^ ion pairs, Mn^2+^ occupying cubically coordinated sites, as well as conjectures positing the involvement of Mn^3+^ oxidized from Mn^2+^ or defects. Despite these diverse and valuable insights, none of the hypotheses have yet achieved broad consensus. In this study, we have observed prolonged fluorescence lifetimes (~10 ms) for the NIR emissions of Mn^2+^ ions, hinting at these ions occupying the high-symmetry octahedral sites inherent to the garnet lattice. This inference is supported by the corroborating results from X-ray absorption fine structure analysis and first-principles calculations. The intense crystal field of octahedral sites, similar to that of AlO_6_, facilitates the splitting of *d*–*d* energy levels, thereby inducing a red-shift in the emission spectrum to the NIR region due to the transition ^4^T_1_(^4^G) → ^6^A_1_(^6^S) of isolated Mn^2+^. Our findings not only offer a plausible rationale for the NIR emission exhibited by other Mn^2+^-activated garnet phosphors but also pave a definitive route towards understanding the fundamental mechanisms responsible for the NIR emission of Mn^2+^ ions.

## Abstract (web summary)

The enduring enigma surrounding the near-infrared (NIR) emission of Mn^2+^ persists, sparking intense academic discussions without reaching a consensus viewpoint.

## Graphical abstract

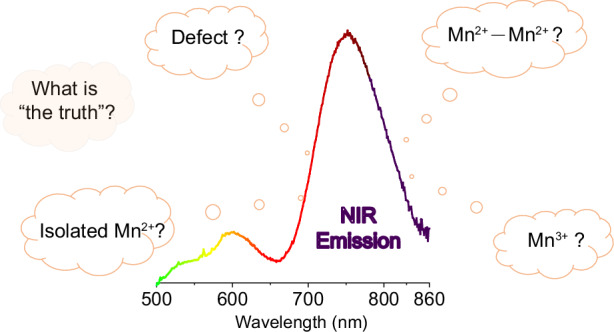

## Introduction

Near-infrared (NIR) light sources, operating within the 700–1000 nm spectrum, have garnered considerable interest across various fields, including biological imaging, non-destructive testing, plant growth illumination, and night vision technologies^1–4^. Recent research efforts have particularly focused on the development of NIR phosphors, with a special emphasis on those that can be efficiently activated by blue light-emitting diodes (LEDs). Once these blue LED-compatible and highly efficient NIR phosphors are realized, the well-established phosphor-converted white-LEDs (pc-WLEDs) technology can be harnessed to miniaturize NIR light sources. This could usher in a new era of intelligent, portable applications while addressing the inefficiencies, bulkiness, and short lifespans of conventional NIR light sources like incandescent bulbs and halogen lamps^1,5–7^.

Currently, the activator ions capable of emitting in the NIR region include rare earth ions (Pr^3+^, Tm^3+^, Yb^3+^, Nd^3+^, Dy^3+^, and Eu^2+^)^6,8–14^, transition metal ions (Ni^2+^, Mn^2+^, Fe^3+^, and Cr^3+^)^1,5–7,15–20^, and bismuth (Bi)^21^. Each of these activators offers distinct advantages, but also comes with inherent limitations, such as a narrow emission spectrum, restricted absorption, or low luminescence efficiency—all of which present significant challenges to overcome. Among these, Cr^3+^ ions remain a preferred option since they are capable of absorbing blue light (^4^A_2g_ → ^4^T_1g_(^4^F)) and emitting broadband NIR light (^4^T_2g_(^4^F) → ^4^A_2g_(^4^F)) when positioned at octahedra sites with a weak crystal field strength. In recent years, considerable research endeavors have been directed towards the development of high-performance NIR phosphors centered on Cr^3+^, yielding continuous progress, especially in boosting internal and external quantum efficiencies (IQE/EQE). Notable examples^1,22–25^ include Ca_3_Sc_2_Si_3_O_12_: Cr^3+^ (*λ*_ex_ = 460 nm, *λ*_em_ = 478 nm, IQE/EQE = 92.3%/25.5%), Y_3_Ga_3_MgSiO_12_:Cr^3+^ (*λ*_ex_ = 438 nm, *λ*_em_ = 782 nm, IQE/EQE = 79.9%/33.7%), Gd_3_Ga_4_AlO_12_:Cr^3+^ (*λ*_ex_ = 448 nm, *λ*_em_ = 730 nm, IQE/EQE = 85%/44%), and Mg_4_Ta_2_O_9_:Cr^3+^ (*λ*_ex_ = 448 nm, *λ*_em_ = 730 nm, IQE/EQE = 87.5%/61.25%). Nevertheless, these NIR emissions arise from partially forbidden *d*–*d* transitions of Cr^3+^, characterized by a small absorption cross-section (ranging from 10^−19^ to 10^−20^ cm^2^)^2,26,27^, thereby posing a challenge in achieving exceptionally high EQE for NIR phosphors. In contrast, the rare earth Ce^3+^ ion benefits from spin-allowed 4*f*–5*d* transitions, facilitating the development of phosphors with exceptionally high quantum efficiencies (QEs). This is exemplified by the commercially successful yellow phosphor Y_3_Al_5_O_12_:Ce^3+^ (YAG:Ce^3+^), which boasts an EQE of up to 70%^28^.

The most widely utilized commercial pc-WLEDs was produced using an InGaN-based blue LEDs chip that was coated with the yellow phosphor YAG:Ce^3+^. Nevertheless, the pc-WLEDs possesses inherent limitations, primarily the absence of red emission components, which results in white light exhibiting high correlated color temperatures (CCT > 4500 K) and low color rendering indexes (Ra < 80)^29^. In recent years, extensive research efforts have been devoted to the design of innovative red phosphors that harness the Ce^3+^–Mn^2+^ energy transfer. The robust absorption of visible light exhibited by Ce^3+^ effectively addresses the challenges posed by the weak absorption capabilities of Mn^2+^ ions^27^. Furthermore, unlike the Ce^3+^–Cr^3+^ system^30,31^, the Ce^3+^–Mn^2+^ system is devoid of luminescence quenching traps, rendering it a highly appealing candidate for enhancing the red emission component in pc-WLEDs. If the existence of Mn^2+^-activated NIR emission is verified, the establishment of an energy transfer from Ce^3+^ to Mn^2+^, along with the precise manipulation of the local crystal field surrounding Mn^2+^, could pave the path towards the development of highly efficient NIR phosphors. However, a fundamental comprehension of the origins underlying NIR emission for Mn^2+^ remains an essential prerequisite for attaining this objective.

Transition metal ions, specifically Mn^2+^ and Cr^3+^, have played a pivotal role in the progress of luminescent materials. Notably, Mn^2+^ demonstrates an exceptional capacity to emit green, red, and potentially even NIR light, contingent upon its specific local crystal field environment. In phosphors^32–34^ including BaZnAl_10_O_17_:Mn^2+^ (*λ*_ex_ = 450 nm, *λ*_em_ = 517 nm), Sr_2_MgAl_22_O_36_:Mn^2+^ (*λ*_ex_ = 450 nm, *λ*_em_ = 518 nm), and Na_2_MgSiO_4_:Mn^2+^ (*λ*_ex_ = 450 nm, *λ*_em_ = 520 nm), the presence of Mn^2+^ occupying tetrahedral sites contributes significantly to the green emission. Conversely, red emission is discernible when Mn^2+^ ions occupy octahedral sites, as exemplified in compounds^35–37^ like NaScSi_2_O_6_:Mn^2+^ (*λ*_ex_ = 410 nm, *λ*_em_ = 654 nm), Ca_18_K_3_Sc(PO_4_)_14_:Mn^2+^ (*λ*_ex_ = 405 nm, *λ*_em_ = 640 nm), and Na_2_Mg_2_Si_6_O_15_:Eu^2+^, Mn^2+^ (*λ*_ex_ = 365 nm, *λ*_em_ = 630 nm). Nevertheless, the underlying mechanisms responsible for the NIR emission triggered by Mn^2+^ continue to be a subject of intense discussion. Earlier hypotheses ascribed the NIR emission to Mn^2+^ ions occupying cubically coordinated sites^38,39^, whereas contemporary perspectives favor the coupling effect arising from Mn^2+^–Mn^2+^ pairs^16,40–43^. However, Meijerink et al. have expressed skepticism towards the latter perspective^44^, contending that Mn^2+^–Mn^2+^ coupling does not significantly contribute to the red-shifted emissions. Instead, it is hypothesized that defects or the oxidation of Mn^2+^ to Mn^3+^ underlie the observed mechanisms.

Manganese (Mn) exhibits remarkable versatility in assuming a wide range of valence states^45^, ranging from +1 to +7, with Mn^2+^ ions demonstrating a particular proficiency in adapting to local structure characterized by four-, six-, or eight-fold coordination configurations. Notably, a significant number of Mn^2+^-activated NIR phosphors, including MnAl_2_O_4_^16^, *α*-MnS^41^, and KMnF_3_^42^ display a unique behavior where Mn^2+^ ions occupy neighboring sites. These distinguishing features pose a significant hurdle in deciphering the underlying mechanism governing the NIR emission exhibited by Mn^2+^ ions. Fortunately, Mn^2+^ exhibits both NIR and red emissions within the garnet structure, thereby offering an invaluable opportunity to delve deeper into the origins of NIR luminescence in Mn^2+^ ions. Within the garnet structure, we can not only scrutinize the valence states of manganese (Mn) but also rigorously explore the fundamental causes of its NIR and red emissions. Moreover, the establishment of a Mn^2+^–Mn^2+^ model within the garnet structure for first-principles calculations greatly aids in validating the ion pair coupling effects. Leveraging our previous achievements^27^, where the Ce^3+^–Mn^2+^ energy transfer within the garnet facilitated ultra-wideband emission, we have broadened the application spectrum of single-matrix pc-WLEDs. Our ongoing endeavor leverages the venerable garnet-based phosphor YAG:Ce^3+^ as a fundamental building block in establishing a Ce^3+^–Mn^2+^ energy transfer system, ultimately yielding an Mn^2+^-activated garnet phosphor that predominantly emits in the NIR region. The insights gained from our research not only enhance our understanding NIR emission of Mn^2+^ but also pave promising paths for future investigative endeavors aimed at developing NIR phosphors.

## Results

### Phase analysis

**Fig. 1:**
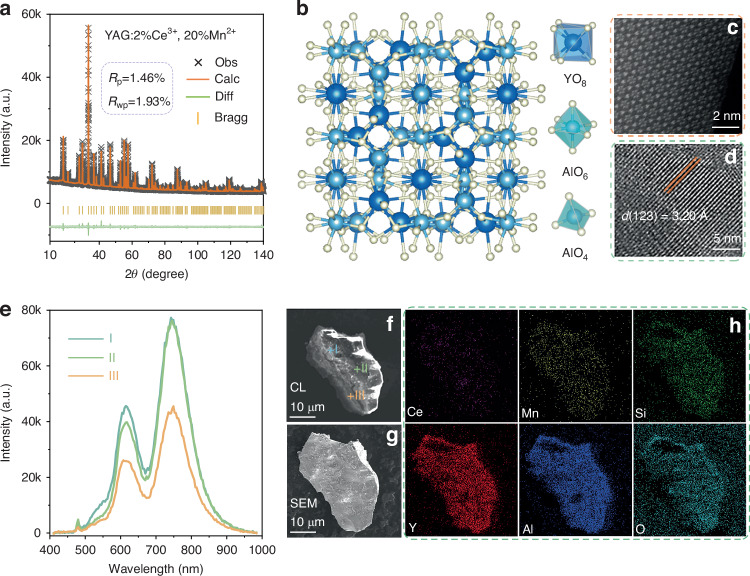
Phase analysis of YAG:2%Ce^3+^, 20%Mn^2+^. **a** Rietveld refinements for XRD pattern; **b** the crystal structure and the coordination environments; **c** STEM–HAADF images; **d** HRTEM images; **e**, **f** CL spectra at points I, II and III and corresponding CL image; **g**, **h** SEM image, and EDS elemental mapping images

The phase purity of YAG:2%Ce^3+^, *x*Mn^2+^, with *x* varying from 0% to 20%, has been extensively analyzed through meticulous XRD analysis and Rietveld refinement. Figure 1a and Table 1, along with Fig. S1 and Table S1 (Supporting Information), provide comprehensive details and understanding of the purity characteristics of these samples. Remarkably, the Rietveld refinement outcomes for YAG doped with 2%Ce^3+^ and 20%Mn^2+^ ions (*R*_p_ = 1.46%, *R*_wp_ = 1.93%, *χ*^*2*^ = 2.12) unequivocally validate the successful integration of Ce^3+^ and Mn^2+^ ions into the YAG crystal lattice. Nevertheless, when the Mn^2+^ content exceeds *x* = 20%, a minor impurity phase appears in the YAG structure, as clearly illustrated in Fig. S2. As a result, our meticulous investigation has narrowed down our focus to the specific composition of YAG:2%Ce^3+^, *x*Mn^2+^, where *x* ranges from 0% to 20%, in order to accurately determine the origin of NIR emission. Furthermore, to facilitate the analysis of the luminescence phenomenon in YAG:2%Ce^3+^, 20%Mn^2+^, we also synthesized YAG:20%Mn^2+^ and Y_2_Mg_0.92_Mn_0.08_Al_4_SiO_12_ for QEs and coordination number analysis, as detailed in the following text. Their diffraction patterns are depicted in Fig. S3.

**Table. 1:** The Rietveld refinement data of YAG:2%Ce^3+^, 20%Mn^2+^

Formula	YAG:2%Ce^3+^, 20%Mn^2+^
Crystal system	${\rm{la}}\bar{3}{\rm{d}}$
Space group	230
*a* = *b* = c (Å)	11.98239
*α* = *β* = *γ* (°)	90
Cell volume	1720.402
*R*_p_(%)	1.46%
*R*_wp_(%)	1.93%
*χ*^2^	2.12

YAG exhibits a cubic Ia $\bar{3}$ d symmetry, as shown schematically in Figs. 1b and S4, encompassing three distinct cation sites: the distorted dodecahedral YO_8_ with C_2_ point symmetry, the octahedral AlO_6_ with C_3d_ point symmetry, and the tetrahedral AlO_4_ with S_4_ point symmetry. These cation sites collaborate to intricately forge the three-dimensional garnet framework through shared edges and vertices. Given the significant doping of Mn^2+^ at a concentration of 20%, high-resolution scanning transmission electron microscopy (STEM), in conjunction with high-angle annular dark-field (HAADF) imaging techniques, was utilized to meticulously investigate the atomic-scale architecture of YAG:2%Ce^3+^, 20%Mn^2+^. As clearly depicted in Fig. 1c, the vivid spots accurately pinpoint the precise locations of cation sites within a highly structured and well-defined garnet lattice, conspicuously free of any perceptible defects.

High-resolution transmission electron microscopy (HRTEM) images offer a revealing glimpse into micrometer-sized YAG:2%Ce^3+^, 20%Mn^2+^ phosphors, exhibiting irregular morphologies as depicted in Fig. S5. Notably, the observed lattice fringe spacing of 3.08 Å, as shown in Fig. 1d, likely corresponds to the (1 2 3) plane of the YAG structure. Figures 1e and S6 comprehensively present cathodoluminescence (CL) spectra, scanning electron microscopy (SEM) imagery, and energy-dispersive X-ray spectroscopy (EDS) elemental mappings of randomly sampled particles extracted from this phosphor. Despite variations in CL intensity, both brightly illuminated regions (I) and those with more muted luminescence (II, III) consistently exhibit a characteristic NIR emission that peaks at approximately 750 nm. The EDS mapping highlights the uniform distribution of Cerium (Ce), Manganese (Mn), silicon (Si), Yttrium (Y), Aluminum (Al), and Oxygen (O) throughout the particle, further corroborating the homogeneity of the doped system.

Collectively, these phase analyses confirm that Ce^3+^ and Mn^2+^ can be seamlessly and uniformly incorporated into the YAG crystal lattice without causing any structural degradation or generating impurities and defects.

### Valence state, steady-state and transient photoluminescence characteristics

**Fig. 2:**
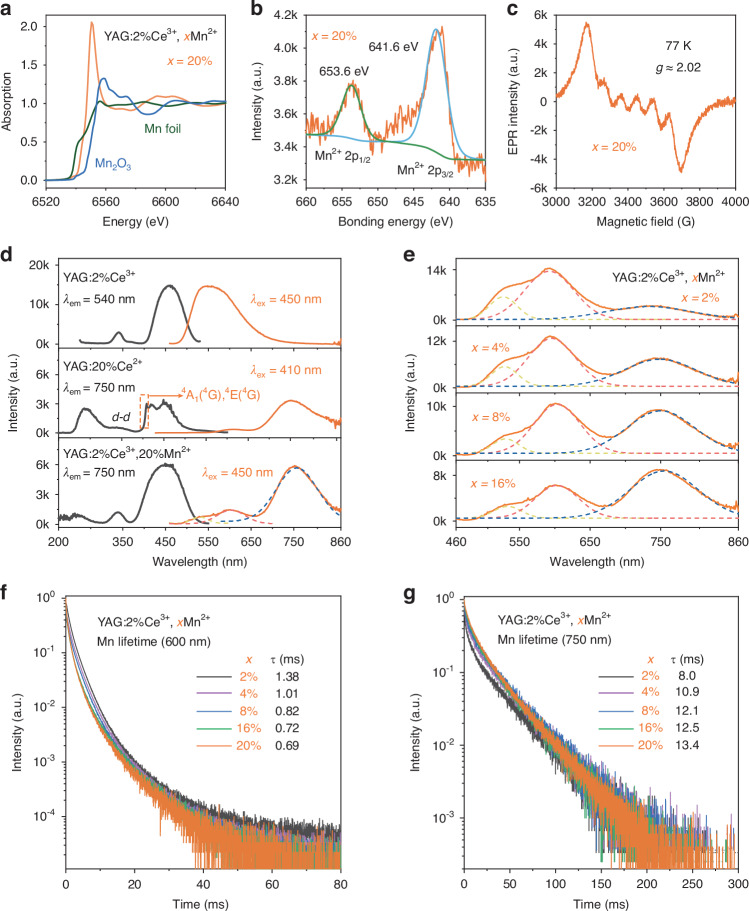
Analysis of the valence state, steady-state and transient photoluminescence characteristics were conducted. **a**–**c** This includes XANES spectra, a high-resolution XPS spectrum for Mn-2p, and EPR spectra recorded at 77 K, all specific to Mn within the sample of YAG doped with 2%Ce^3+^ and 20%Mn^2+^; **d** Additionally, PLE and PL spectra were obtained for YAG doped with 2% Ce^3+^, YAG doped with 20%Mn^2+^, and YAG co-doped with both 2%Ce^3+^ and 20%Mn^2+^; **e** PL spectra were also recorded for YAG doped with 2%Ce^3+^ and varying concentrations of Mn^2+^ (ranging from *x* = 2% to 16%); **f**, **g** Furthermore, the fluorescence decay curves of Mn^2+^ in YAG doped with 2% Ce^3+^ and varying concentrations of Mn^2+^ (*x* = 2% to 20%) upon pulsed 450 nm excitation, monitored at 600 nm and 750 nm, are presented. Detailed fitting equations and parameters for these decay curves are outlined in Tables S2–S4

To unequivocally determine the valence state of Mn within YAG matrix, we employed a comprehensive approach that encompassed X-ray absorption near edge structure (XANES) analysis, X-ray photoelectron spectroscopy (XPS), and electron paramagnetic resonance (EPR) techniques on the YAG:2%Ce^3+^, 20%Mn^2+^ samples. Drawing parallels from our previous inquiries^27^, the valence state of Mn within the Lu_2_BaAl_4_SiO_12_ garnet structures exhibited a striking resemblance to the near-edge absorption pattern characteristic of MnO (Mn^2+^), as revealed through XANES analysis. Similarly, Fig. 2a demonstrates that the Mn absorption edge in YAG is intermediate between the absorption edges of Mn_2_O_3_ (Mn^3+^) and Mn foil, indicating the retention of a positive divalent state for Mn (e.g., Mn^2+^) in the YAG structure.

The XPS spectra in Figs. 2b and S7 exhibit distinct peaks at 653.6 eV and 641.6 eV, respectively. These peaks can be attributed to the characteristic Mn 2p_1/2_ and Mn 2p_3/2_ transitions, which are associated with Mn^2+^ ions^46^. The EPR spectrum in Fig. 2c clearly exhibits a well-resolved sextet pattern, indicative of Mn^2+^, resulting from the transition Ms = |–1/2> to |+1/2>, which is superimposed with the hyperfine interaction between the electron spin and the 55Mn nucleus (*I* = 5/2). Furthermore, the isotropic g-value, determined to be approximately 2.02 based on the spin Hamiltonian (Eq. (S1) in the Supporting Information), suggests the presence of Mn^2+^ ions occupying octahedral symmetry sites within the YAG structure^47^. Moreover, as clearly illustrated in Fig. 2d, the photoluminescence excitation (PLE) spectrum of YAG:20Mn^2+^ prominently displays a characteristic *d*–*d* transition of Mn^2+^ ions, manifested by the NIR emission peak centered around ~750 nm. Remarkably, a narrow band located approximately at ~400 nm arises from the transition of ^6^A_1_^6^(S) to [^4^A_1_(^4^G), ^4^E(^4^G)] states, which is the most prominent attribute of Mn^2+^ ions^48^. Drawing upon the converging evidence from XANES, XPS, EPR, and the spectral findings presented in Fig. 2d, we can unequivocally establish that the valence state of Mn ions in the YAG host lattice is divalent, specifically Mn^2+^.

Generally, the emission of Ce^3+^-activated oxide phosphors primarily occurs within the violet to blue spectral regions^49–51^. Notably, the Ce^3+^-doped YAG phosphor exhibits a distinct characteristic due to the unique positioning of Ce^3+^ within a dodecahedral YO_8_ site. In this configuration, the substantial overlap of electron clouds from the eight surrounding oxygen ions significantly enhances the Ce–O covalency, resulting in a pronounced centroid shift. Furthermore, the distorted YO_8_ configuration significantly enhances the splitting between the e_g_ and t_2g_ energy levels of Ce^3+^ in comparison to octahedral or cubic configurations, thereby facilitating the robust absorption of blue light and emission of yellow light in YAG:Ce^3+^^51^. Figure 2d illustrates comparative PLE and photoluminescence (PL) spectra for YAG doped with 2%Ce^3+^, 20%Mn^2+^, and a combination of 2%Ce^3+^ and 20%Mn^2+^. Specifically, YAG doped with 2%Ce^3+^ exhibits a prominent yellow emission peak centered precisely at 540 nm, arising from transitions involving the lowest 5*d* state of Ce^3+^ to its ^2^F_5/2_ and ^2^F_7/2_ energy levels. Notably, the significant overlap observed between the PL spectrum of Ce^3+^ and the PLE spectrum of Mn^2+^ suggests a potential energy transfer from Ce^3+^ to Mn^2+^ within the YAG lattice^52^. Upon excitation of Ce^3+^ ions at approximately 450 nm within the YAG:2%Ce^3+^, 20%Mn^2+^ phosphor, dual emissions are observed: one emanating from Ce^3+^ at approximately 540 nm and another originating from Mn^2+^ at approximately 600 nm and 750 nm. Furthermore, as illustrated in Figs. S8, S9, Table S2 and Eqs. (S2)–(S4) in the Supporting Information, the fluorescence lifetime of Ce^3+^ decreases gradually from 56 ns (at *x* = 0) to 8 ns (at *x* = 20%). These observation confirm the occurrence of energy transfer from Ce^3+^ to Mn^2+^ and suggests that the energy transfer efficiency (*η*_T_) approaches 86% at *x* = 20% within the YAG matrix. Thanks to the assistance of Ce³⁺ ions, Mn²⁺ effectively captures blue light (~450 nm), achieving an absorption efficiency (AE) of up to 60%. As shown in Fig. 2e, at lower Mn^2+^ doping concentrations ranging from 2% to 8%, the primary Mn^2+^ emission in YAG is predominantly situated in the red region (~600 nm). Importantly, when the Mn^2+^ doping level exceeds 16%, the NIR emission (~750 nm) gradually becomes the dominant feature in the PL spectrum. To more clearly illustrate the impact of Mn^2+^ doping (*x*) on the energy distribution of emission spectra, we conducted peak fitting on the emission spectra of YAG:2%Ce^3+^, *x*Mn^2+^ (where *x* ranges from 2% to 20%). Additionally, as demonstrated in Figs. 2d, e and S10, we calculated the proportions of the spectral integral intensities of Ce^3+^ (~540 nm), Mn^2+^ (~600 nm), and Mn^2+^ (~750 nm) within the overall emission spectrum.

Within the context of YAG:Ce^3+^, Mn^2+^, a prime opportunity emerges to delve deeper into the genesis of NIR emission emanating from Mn^2+^ ions. Initially, it is unlikely that the NIR emission originates from the coupling of Mn^2+^–Mn^2+^ ion pairs, as the generation of Mn^2+^–Mn^2+^ pair effects leading to the transition |^6^A_1_,^4^T_1_> → |^6^A_1_,^6^A_1_> typically necessitates the doping of higher concentrations of Mn^2+^ ions into the crystal lattice^16,40–43^. As shown in Fig. 2e, it is evident that the NIR emissions from Mn^2+^ ions are discernible even at a minimal doping concentration of 2%. Typically, during the preparation stage, raw materials enriched with Mn^2+^ undergo meticulous grinding procedures before being subjected to high-temperature calcination. This process results in Mn^2+^ being disseminated throughout the YAG lattice in a random yet uniform manner. Consequently, at lower concentrations of doping, the occurrence of Mn^2+^–Mn^2+^ ion pairing diminishes notably, suggesting that the observed NIR emission in Mn^2+^-doped YAG is unlikely to stem from the interaction between Mn^2+^–Mn^2+^ ion pairs. We can also categorically eliminate Mn^3+^ as the source of NIR emission by extending the PL spectrum of YAG:2%Ce^3+^, 20%Mn^2+^ across the 500 to 1200 nm range. The most distinguishing characteristic of Mn^3+^ emissions within the garnet structure is the intense luminescence arising from the transition ^1^T_2_ → ^3^T_2_, which is centered precisely between 1100 nm and 1200 nm^53^. It is noteworthy that, as clearly depicted in Fig. S11, both the spectra of YAG:2%Ce^3+^, 20%Mn^2+^ at room temperature (RT) and at 77 K exhibit a distinct absence of detectable luminescence within the crucial 1100 nm to 1200 nm region, thus firmly validating our initial conclusion.

The time-resolved spectroscopic analysis of YAG doped with Ce^3+^ and Mn^2+^ ions reveals a strikingly rapid decay rate for the red emission (~590 nm). As clearly demonstrated in Fig. S12, following a 10 ms delay, only the NIR emission centered at approximately 750 nm remains, indicating that these two emissions originate from distinct luminescent centers within the YAG crystal structure^54,55^. Remarkably, as shown in Fig. 2f, g, the NIR emission demonstrates a fluorescence lifetime of approximately ~10 ms, significantly surpassing the ~1 ms observed for the red emission (~590 nm). This suggests that the NIR emission cannot be attributed to Mn^2+^–Mn^2+^ coupling within the red emission center, as such coupling is expected to relax the spin selection rule and thus shorten the fluorescence lifetime (see Fig. S13 and Eq. (S5))^56^. More precisely, the extended NIR emission lifetime (~10 ms) indicates Mn^2+^ residing in high-symmetry positions within the garnet crystal structure. As schematically illustrated in Figs. 1b and S4, the 8-fold coordination gives rise to a distorted dodecahedral YO_8_ configuration, whereas the 6-fold coordination corresponds to an octahedral AlO_6_ site, and the 4-fold coordination presents a tetrahedral AlO_4_ arrangement. By leveraging previous research that employed synchrotron radiation to elucidate the Mn^2+^ coordination in garnet structures, we know that Mn^2+^ ions occupy both 8- and 6-coordinated sites^27^. Therefore, we hypothesize that the NIR emissions shown in Fig. 2d, e originate from these Mn^2+^ ions residing within 6-coordinate octahedral AlO_6_ sites. As illustrated in Fig. S14, we believe that the transitions ^4^T_1_(^4^G) → ^6^A_1_(^6^S) of isolated Mn^2+^ ions occupying octahedral sites not only result in the red emission but potentially also initiate an NIR emission, attributed to the intensified splitting of *d*–*d* energy levels due to by the intense local crystal field of AlO_6_ configurations characterized by shorter Al–O bonds. Regarding the origin of the red emission (~600 nm) exhibited by YAG:Ce^3+^, Mn^2+^, we postulate that it arises from Mn^2+^ ions residing within the distorted dodecahedral YO_8_ sites. Despite the elongation of the Y–O bonds, the distortion of the YO_8_ environment fosters a robust crystal field, thereby enabling Mn^2+^ to emit red light upon occupancy. This mechanism is analogous to that observed in Ce^3+^ occupies the YO_8_ site of YAG structure, resulting in yellow emission^51^. In addition, the factors pertaining to the crystal field strength are mirrored in Eq. (S6) of the Supporting Information.

### Forecasting spectroscopic characteristics through first-principles calculations

**Fig. 3:**
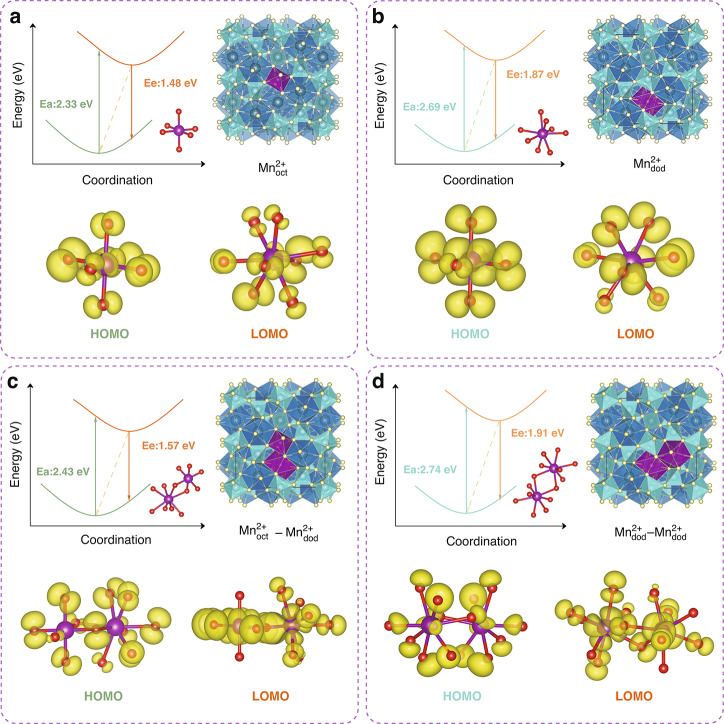
First-principles calculations for YAG:2%Ce^3+^, 20%Mn^2+^. Configuration coordinate diagrams for the ground state ^6^A_1_ and excited states ^4^T_1_ of Mn^2+^-doped YAG structure, along with the corresponding partial charge density distribution of the highest occupied orbital and the lowest unoccupied orbital, are presented. In this context, four distinct computational models are derived from the local structure of YAG. **a** Isolated Mn^2+^ ion occupies octahedron site (${\rm{Mn}}^{2+}_{\rm{oct}}$); **b** Isolated Mn^2+^ ion occupies dodecahedron site (${\rm{Mn}}^{2+}_{\rm{dod}}$); **c** Mn^2+^ ions occupy the adjacent octahedron and dodecahedron sites (${\rm{Mn}}^{2+}_{\rm{oct}}{-}{\rm{Mn}}^{2+}_{\rm{dod}}$); **d** Mn^2+^ ions occupy the adjacent two dodecahedron sites (${\rm{Mn}}^{2+}_{\rm{dod}}{-}{\rm{Mn}}^{2+}_{\rm{dod}}$)

First-principles calculations are recognized as a robust method for forecasting the energy level structure and spectroscopic characteristics of Mn^2+^ ions^57^. To validate our hypothesis, as illustrated in Figs. 3, S15 and Table S5, we constructed four distinct computational models based on the local structure of YAG. These models encompass: an individual Mn^2+^ ion occupying an isolated octahedral site (Fig. 3a), designated as ${\rm{Mn}}^{2+}_{\rm{oct}}$; a solitary Mn^2+^ ion residing within a dodecahedral coordination sphere (Fig. 3b), labeled as ${\rm{Mn}}^{2+}_{\rm{dod}}$; a scenario wherein octahedral sites are adjacent to dodecahedral sites, creating an interconnected environment (${\rm{Mn}}^{2+}_{\rm{oct}}{-}{\rm{Mn}}^{2+}_{\rm{dod}}$, Fig. 3c); and finally, a configuration featuring dodecahedral sites alongside their geometrically equivalent neighboring sites (${\rm{Mn}}^{2+}_{\rm{dod}}{-}{\rm{Mn}}^{2+}_{\rm{dod}}$, Fig. 3d). Our calculations reveal the emission energies of the ^4^T_1_ → ^6^A_1_ transition for Mn^2+^ in isolated octahedral (${\rm{Mn}}^{2+}_{\rm{oct}}$) and dodecahedral (${\rm{Mn}}^{2+}_{\rm{dod}}$) sites are 1.48 eV and 1.87 eV, respectively. Notably, first-principles analysis underscores that the NIR emission in YAG:Mn^2+^ originates from Mn^2+^ ions occupying octahedral AlO_6_ sites (${\rm{Mn}}^{2+}_{\rm{oct}}$), while the red emission emanates from Mn^2+^ occupying distorted dodecahedral YO_8_ sites (${\rm{Mn}}^{2+}_{\rm{dod}}$). However, contrary to the prevalent belief, our findings do not corroborate the notion that Mn^2+^–Mn^2+^ pairs contribute to NIR emission in the YAG structure. The electronic environments surrounding these pairs closely resemble those observed in isolated ${\rm{Mn}}^{2+}_{\rm{oct}}$ and ${\rm{Mn}}^{2+}_{\rm{dod}}$ configurations. Furthermore, the emission energies of Mn^2+^–Mn^2+^ pairs, estimated at 1.57 eV for ${\rm{Mn}}^{2+}_{\rm{oct}}$ and 1.91 eV for ${\rm{Mn}}^{2+}_{\rm{dod}}$ in pair system, closely align with those of their isolated counterparts, emphasizing their similarity. While previous studies on Mn^2+^–Mn^2+^ pairs has shown that these paired ions can modulate the absorption spectra, fluorescence lifetime, and other characteristics of Mn^2+^^56,58^. There remains a lack of conclusive direct evidence to support a significant red-shift in the emission spectrum caused by Mn^2+^–Mn^2+^ pairs in the YAG structure.

### XAS characterizations

**Fig. 4:**
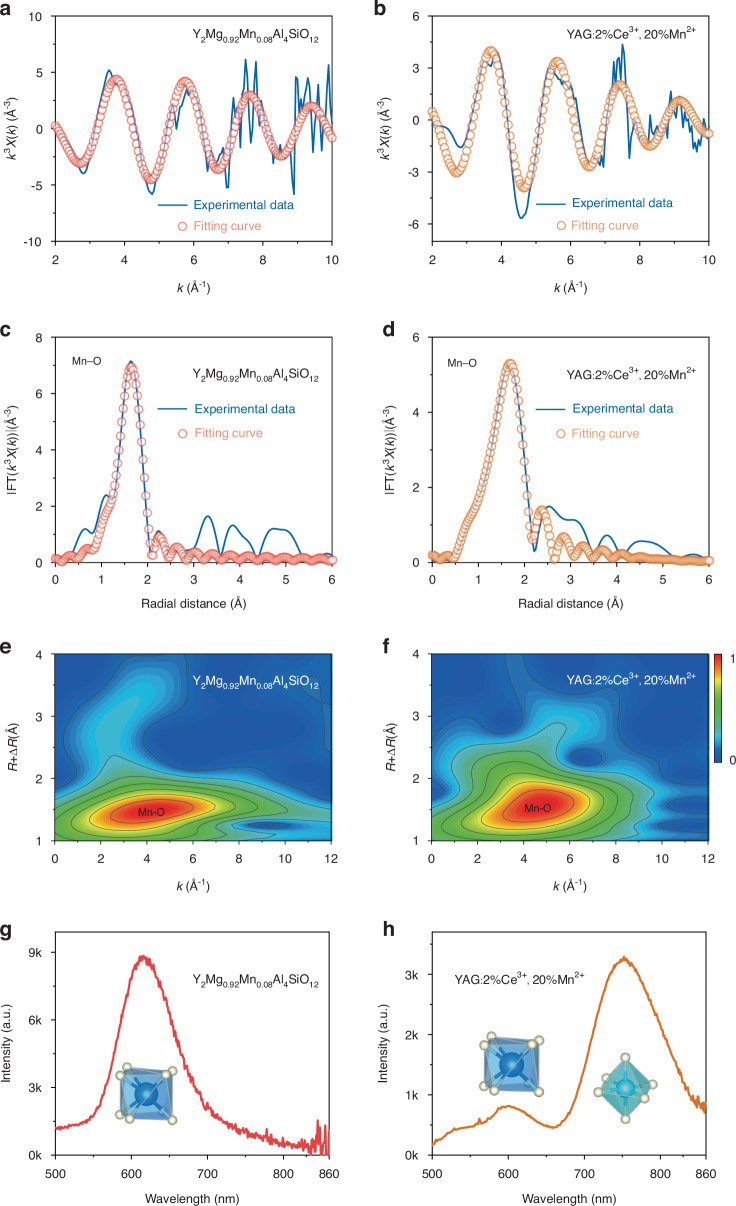
XAS characterizations of Y_2_Mg_0.92_Mn_0.08_Al_4_SiO_12_ and YAG:2%Ce^3+^, 20%Mn^2+^. **a**, **b** FT-EXAFS spectra; **c**, **d** Experimental and calculated EXAFS spectra χ(*k*)*k*^2^; **e**, **f** Wavelet transform of the *k*^3^-weighted EXAFS data; **g**, **h** Photoluminescence and corresponding coordination environments

X-ray absorption spectroscopy (XAS) provides an exceptional method for unveiling the intricate structural details of Mn^2+^ within the garnet structure, including its valence state, coordination number, bond length, and other relevant parameters. Our group’s prior investigations have unequivocally determined that the average coordination number of Mn^2+^ in the garnet structure is seven, indicating its presence in both six- and eight-coordinated sites^27^. This leads us to envision the creation of Mn^2+^-activated garnet structures tailored to exclusively exhibit either red or NIR emission centers. Then, through meticulous XAS analysis of their coordination numbers, we can directly pinpoint the origin of NIR emission in Mn^2+^-activated garnet structures. Fortunately, as shown in Fig. 2e, Mn^2+^ exhibits a preferential propensity for forming red luminescent centers in garnet structure. In this pursuit, as depicted in Fig. S3, we have achieved a successful substitution of Y^3+^–Al^3+^ in YAG lattice with Mg^2+^–Si^4+^. Additionally, we further substituted Mg^2+^ with Mn^2+^, ultimately yielding a Mn^2+^-activated garnet phosphor (Y_2_Mg_0.92_Mn_0.08_Al_4_SiO_12_) that exclusively exhibits red emission. The extended X-ray absorption fine structure (EXAFS) spectra, accompanied by fitting data for both Y_2_Mg_0.92_Mn_0.08_Al_4_SiO_12_ and YAG:2%Ce^3+^, 20%Mn^2+^, are presented in Fig. 4a–f, with corresponding data are detailed in Tables 2, S6 and Figs. S16, S17. It is noteworthy that the fitting outcomes attribute the red luminescence of Y_2_Mg_0.92_Mn_0.08_Al_4_SiO_12_ to Mn^2+^ occupying an 8-coordinate site (${\rm{Mn}}^{2+}_{\rm{dod}}$). Consequently, the dual red and NIR emission centers observed in YAG:2%Ce^3+^, 20%Mn^2+^ can be traced to Mn^2+^ occupying 8- and 6-coordinate sites, respectively, as illustrated in Fig. 4g, h and Table 2. It is crucial to note that the majority of Mn^2+^-activated NIR phosphors, as exemplified by MgAl_2_O_4_:Mn^2+^, ZnAl_2_O_4_:Mn^2+^ and LaZnAl_11_O_19_:Mn^2+^ in Fig. S18^16,40,43,44^, share a commonality in their octahedral sites. This suggests that the NIR emission of Mn^2+^ is attributed to its occupancy within octahedral sites (${\rm{Mn}}^{2+}_{\rm{oct}}$), which are characterized by strong crystal field strength. This provides fundamental insights into the mechanism behind this intriguing phenomenon.

**Table 2:** EXAFS fitting parameters at the Mn K-edge for Y_2_Mg_0.92_Mn_0.08_Al_4_SiO_12_ and YAG:2%Ce^3+^, 20%Mn^2+^ (Ѕ_0_^2^=0.718)

Sample	Y_2_Mg_0.92_Mn_0.08_Al_4_SiO_12_	YAG:2%Ce^3+^, 20%Mn^2+^
Shell	Mn–O	Mn–O
^a^CN	**8.3** **±** **0.2**	**6.9** **±** **0.1**
^b^R(Å)	2.132 ± 0.011	2.168 ± 0.032
^c^σ^2^ (Å^2^)	0.0071 ± 0.0015	0.0112 ± 0.0013
^d^ΔE_0_(eV)	−3.4 ± 1.8	−1.9 ± 1.1
R factor	0.0058	0.0163

^a^CN, coordination number

^b^R, the distance to the neighboring atom

^c^σ^2^, the Mean Square Relative Displacement (MSRD)

^d^ΔE_0_, inner potential correction; R factor indicates the goodness of the fit. S_0_^2^ was fixed to 0.718, according to the experimental EXAFS fit of Mn foil by fixing CN as the known crystallographic value

*During the EXAFS fitting, these values were fixed based on the known structure of Mn. Fitting range: 2.0 ≤ k(Å) ≤ 9.5 and 1.0 ≤ R(Å) ≤ 2.5 (YAG:2%Ce^3+^, 20%Mn^2+^); 3.0 ≤ k(Å) ≤ 10.0 and 1.0 ≤ R(Å) ≤ 2.5 (Y_2_Mg_0.92_Mn_0.08_Al_4_SiO_12_). A reasonable range of EXAFS fitting parameters: 0.700 < Ѕ_0_^2^ < 1.000; CN > 0; σ^2^ > 0 Å^2^; |ΔE_0_| < 10 eV; R factor < 0.02

### Luminescence properties of the fabricated NIR pc-LEDs

**Fig. 5:**
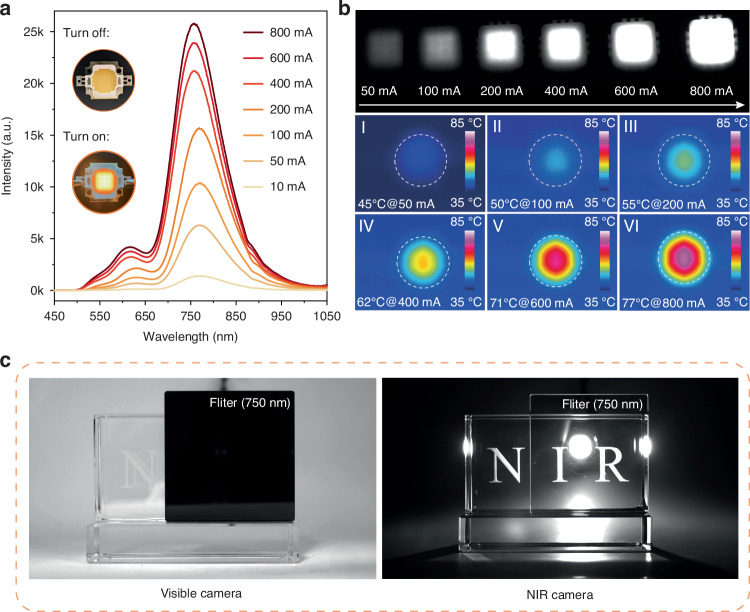
Luminescence properties of NIR pc-LEDs using YAG:2%Ce^3+^, 20%Mn^2+^. **a** Emission spectra of the fabricated pc-LEDs at varied current over 10–800 mA; **b** NIR images and thermal images of the fabricated pc-LEDs at varied driving currents; **c** Photos of the lighting pc-LEDs taken by a normal camera and a NIR camera

We have successfully crafted a NIR pc-LEDs device by seamlessly integrating a NIR phosphor, specifically YAG:2%Ce^3+^, 20%Mn^2+^, with an InGaN blue-LED chip emitting at approximately 450 nm, as depicted in Fig. 5a. The accompanying inset photograph showcases the fabricated pc-LEDs in both idle and activated states. Notably, the emission intensity of the device increases with the gradual rise in injected current. As documented in Fig. S19, precise measurements reveal that the NIR output power is 36.5 mW at 800 mA, while the photoelectric efficiency is 2.4% at 10 mA. Furthermore, a thermal imaging system was utilized to meticulously track the temperature fluctuations of the pc-LEDs device as the injection current increased. As shown in Fig. 5b, the device’s temperature steadily increased, rising from 45 °C at 50 mA to 77 °C at 800 mA. It is remarkable that the NIR emission of YAG:2%Ce^3+^, 20%Mn^2+^ demonstrates outstanding stability with negligible shifts even during temperature hikes, maintaining approximately 66% of its ambient temperature luminescence intensity even at 100 °C, as illustrated in Fig. S20. Moreover, the potential of this device in practical applications, such as night vision systems, detection technologies, and biological tissue imaging, has been preliminarily verified, as shown in Figs. 5c and S21. Under dim lighting conditions, the NIR camera, enhanced by our pc-LEDs, effortlessly captures intricate details of optical materials, identifies foreign bodies within fruits, and enables high-fidelity vascular imaging of the palm. These tasks are proven to be beyond the capabilities of a visible-light camera, even under bright illumination. Despite the residual presence of the red and far-red regions in the luminescence spectrum of YAG:Ce^3+^, Mn^2+^, Fig. S22 demonstrates a significant red shift of ~10 nm in the NIR emission of Lu_3_Al_5_O_12_:Ce^3+^, Mn^2+^ (LuAG:Ce^3+^, Mn^2+^) compared to YAG:Ce^3+^, Mn^2+^, which underscores the immense potential for spectral tuning and optimization of Mn^2+^-based materials. A precise and comprehensive understanding of the NIR emissions originating from Mn^2+^ ions holds the key to unlocking advanced NIR materials centered around the Mn^2+^ ion.

## Discussions

In conclusion, we have successfully synthesized the YAG:2%Ce^3+^, 20%Mn^2+^ phosphor, which exhibits a dominant emission within the NIR region, specifically spanning the range of 700 to 900 nm. Preliminary validation suggests the feasibility of incorporating YAG:2%Ce^3+^, 20%Mn^2+^ into NIR pc-LED devices, underscoring its promising potential for future applications. A thorough analysis confirms that within the garnet structure, Mn exists in its divalent (+2) form, and the NIR emission arises from Mn^2+^ occupying 6-coordinate octahedral AlO_6_ sites (${\rm{Mn}}^{2+}_{\rm{oct}}$). Our hypothesis, which posits that Mn^2+^ ions occupying octahedral sites (${\rm{Mn}}^{2+}_{\rm{dod}}$) within a robust crystal field initiates NIR emission, provides a compelling explanation for the NIR phosphors observed in other Mn^2+^-activated materials. This, in turn, offers valuable insights into the underlying mechanisms responsible for NIR emission in Mn^2+^ ions. It is noteworthy that, while the NIR emission of YAG:Ce^3+^, Mn^2+^ only slightly encroaches into the far-red region, the NIR emission exhibited by Mn^2+^ demonstrates significant potential for spectral redshift through modulation of the local crystal field. A comprehensive understanding of the NIR emission capabilities generated by Mn^2+^ bodes well for the future advancement of NIR phosphors.

## Materials and methods

### Synthesis

A series of samples, Y_3_Al_5_O_12_(YAG):2%Ce^3+^, *x*Mn^2+^ (*x* = 0%–20%), were synthesized by substituting Mn^2+^–Si^4+^ for Y^3+^–Al^3+^ in YAG:2%Ce^3+^. To conduct a more in-depth study on the occupancy of Mn^2+^ and its NIR emission characteristics, we also successfully prepared three materials: YAG:20%Mn^2+^, Y_2_Mg_0.92_Mn_0.08_Al_4_SiO_12_, and Lu_3_Al_5_O_12_:2%Ce^3+^, 20%Mn^2+^ (referred to as LuAG:Ce^3+^, Mn^2+^). These materials are prepared using the traditional high-temperature solid-state reaction. The raw materials, Y_2_O_3_(99.99%), Lu_2_O_3_(99.99%), Al_2_O_3_(99.9%), SiO_2_(99.99%), MgO(99.9%), CeO_2_(99.99%) and MnCO_3_(99.9%), were weighed according to the stoichiometric ratio, and ground in an agate mortar adequately to get the mixture. Then, put the mixture into a tube furnace and sintered at 1550 °C for 4 h in the 10% H_2_ + 90% N_2_ reducing atmosphere. The final product was ground into fine powders for the subsequent investigations.

### Characterizations

The Rietveld refinement of X-ray diffraction (XRD) pattern was performed by FullProf program using data collected in multifunctional horizontal X-ray diffractometer (Ultima IV, Rigaku, Japan) with Cu Kα radiation (*λ* = 1.5406 Å). The counting time for each step was set at 1 s with a step size of 0.02. Electron paramagnetic resonance (EPR) spectra was recorded at room temperature on a Bruker Elexsys E580 spectrometer operating at 9.48 GHz. The high-resolution transmission electron microscopy (HRTEM) images and elemental distributions were measured on a transmission electron microscope (JEM-2100F, UHR) and JSM-7600F (JEOL), respectively. The photoluminescence (PL) and photoluminescence excitation (PLE) were recorded by a Hitachi F-7000 and an Edinburg FLS-980 fluorescence spectrophotometer. The temperature-dependent PL spectra were obtained by the measurement system containing using a heating stage (Linkam THMS-600) and a QEPro high performance spectrometer (Ocean Optics) which gives the time-integrated intensities. A pulsed laser from an optical parametric oscillator (OPO) and the electric signal detected by a Tektronix digital oscilloscope TDS 3052 were used to measure the fluorescence decay of Mn^2+^ emission. The IQE was obtained with an absolute photoluminescence quantum yield measurement system (Quantaurus-QY Plus C13534-12, Hamamatsu Photonics).

### X-ray absorption spectroscopy (XAS) measurement and analysis

Data reduction, data analysis, and EXAFS fitting were performed and analyzed with the Athena and Artemis programs of the Demeter data analysis packages that utilizes the FEFF6 program to fit the EXAFS data^59^. The energy calibration of the sample was conducted through a standard Mn foil, which as a reference was simultaneously measured. A linear function was subtracted from the pre-edge region, then the edge jump was normalized using Athena software. The *χ*(*k*) data were isolated by subtracting a smooth, third-order polynomial approximating the absorption background of an isolated atom. The k^3^-weighted *χ*(*k*) data were Fourier transformed after applying a Hanning window function (Δ*k* = 1.0). For EXAFS modeling, the global amplitude EXAFS (CN, R, *σ*^2^ and Δ*E*_0_) were obtained by nonlinear fitting, with least-squares refinement, of the EXAFS equation to the Fourier-transformed data in R-space, using Artemis software, EXAFS of the Mn foil is fitted and the obtained amplitude reduction factor S_0_^2^ value (0.718) was set in the EXAFS analysis to determine the coordination numbers (CNs) in the Mn–O scattering path in sample.

### Computational details

All the calculations are performed in the framework of the density functional theory with the projector augmented plane-wave method, as implemented in the Vienna ab initio simulation package^60^. The generalized gradient approximation proposed by Perdew-Burke-Ernzerhof (PBE) is selected for the exchange-correlation potential^61^. The cut-off energy for plane wave is set to 500 eV. The energy criterion is set to 10^−5^ eV in the iterative solution of the Kohn-Sham equation. All the structures are relaxed until the residual forces on the atoms have declined to less than 0.02 eV/Å. The Franck-Condon approximation is commonly used to interpret the excitation spectrum, that is, assuming that the electronic transition is very fast compared with the motion of nuclei in the lattice^62^.

### Fabrication of the pc-LED devices

The pc-LED devices were fabricated by the GaN-based blue LEDs coated with the mixture which prepared by the synthesized phosphor and transparent silicon resin. The curing process was performed in an oven at 120 °C for 120 min. The photoelectric properties of the fabricated pc-LEDs, such as NIR output power and photoelectric efficiency, were measured using an integrated test system (LHS-1000, EVERFINE) equipped with a high accuracy array spectrophotometer (350–1100 nm, HAAS-2000).

## Supplementary information

Supplementary Information

## Data Availability

The data that support the findings of this study are available from the corresponding author upon reasonable request.
