# Supplementary material for: Tracing the origin of near-infrared emissions emanating from manganese (II)
Source: Light Sci Appl. 2025 May 13;14:194. doi: 10.1038/s41377-025-01816-y (PMC12075802; doi:10.1038/s41377-025-01816-y)
Supplement: Supplementary file 1 — Supplementary Information [file 41377_2025_1816_MOESM1_ESM.docx]

**Supplementary Information**

**Tracing the Origin of Near-Infrared Emissions Emanating from Manganese (II)**

Yu Xiao^1,2^, Xun Yang^3^, Hao-Ran Zhao^1^, Dan Wu^4^, Ming-Xing Chen^5^, Tianxiang Zheng^2^, Rui Zhang^6^, Ling-Dong Sun^1^*, and Chun-Hua Yan^1^*

^1^Beijing National Laboratory for Molecular Sciences, State Key Laboratory of Rare Earth Materials Chemistry and Applications, PKU-HKU Joint Laboratory in Rare Earth Materials and Bioinorganic Chemistry, College of Chemistry and Molecular Engineering, Peking University, Beijing 100871, P. R. China

^2^College of Science, Nanjing Forestry University, Nanjing 210037, P. R. China

^3^College of Materials Science and Engineering, Nanjing Forestry University, Nanjing 210037, P. R. China

^4^School of Physical Science and Technology, Inner Mongolia Key Lab of Nanoscience and Nanotechnology, Inner Mongolia University, Hohhot 010021, P. R. China

^5^Analytical Instrumentation Center of Peking University, Beijing 100871, P. R. China

^6^School of Chemical and Environmental Engineering, Shanghai Institute of Technology, Shanghai, 201418, P. R. China

**This PDF file includes:**

Figures S1 to S22

Tables S1 to S6

Formulas S1 to S6 **Supplementary Figures**


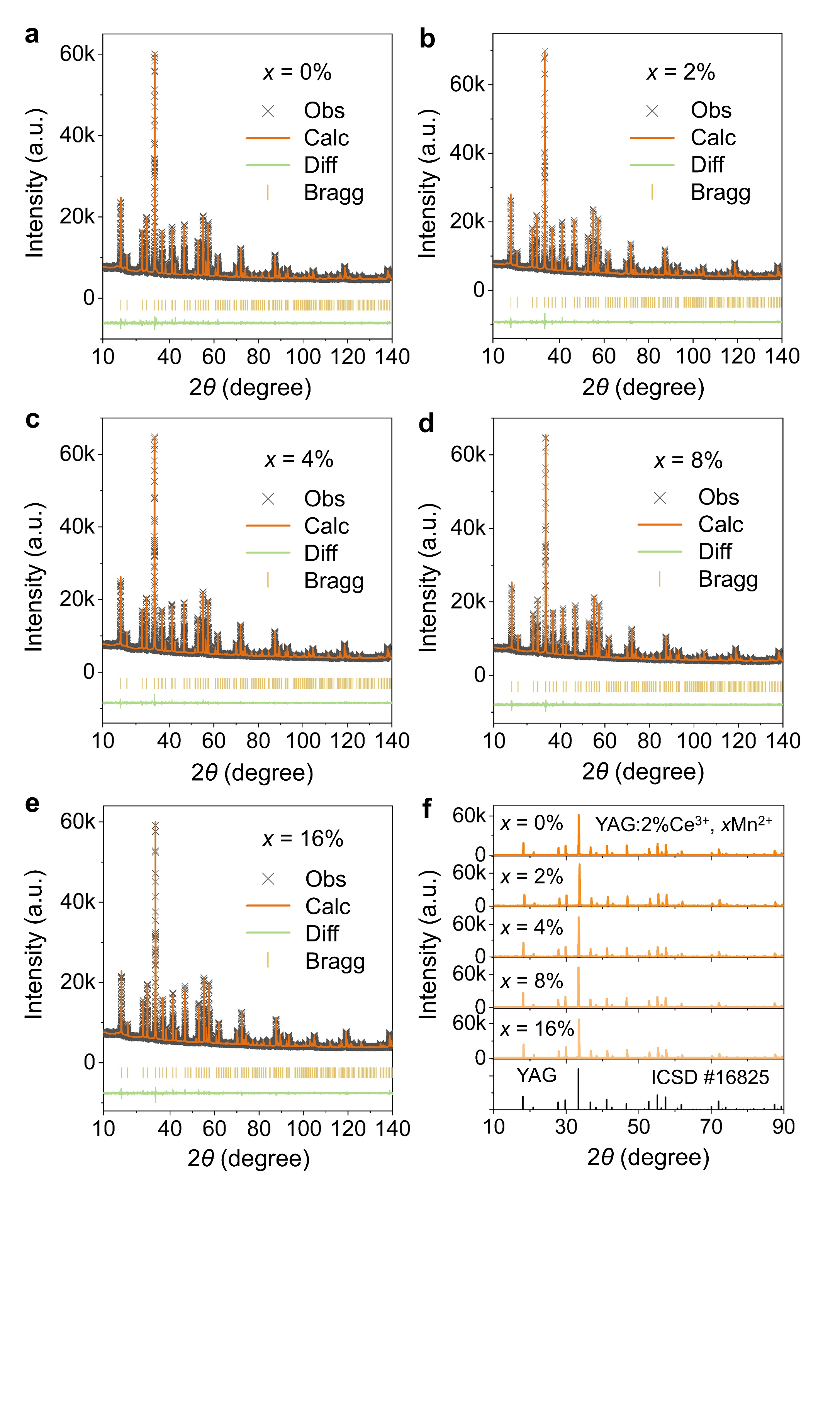


**Fig. S1** **a–e** Rietveld refinement and **f** XRD patterns for YAG:2%Ce^3+^, *x*Mn^2+^ (*x* = 0%–16%). The outcomes of the Rietveld refinement for YAG doped with 2% Ce^3+^ and *x*% Mn^2+^ (*x* = 0%–20%) ions unequivocally confirm the successful integration of Ce^3+^ and Mn^2+^ ions into the YAG crystal lattice without inducing any impurity phase. For a detailed overview of the refinement parameters, please refer to Tables 1 and S1.


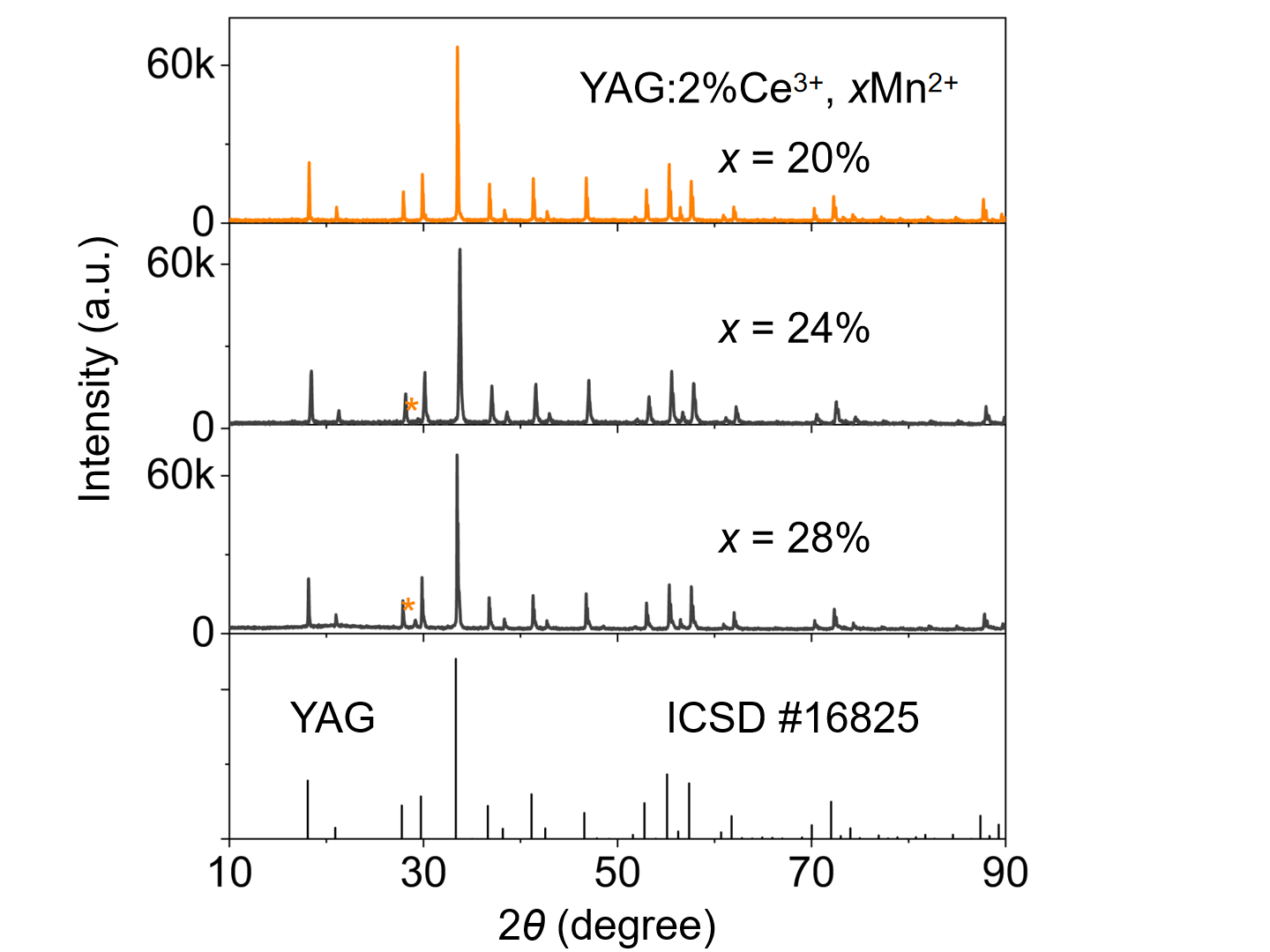


**Fig. S2** The X-ray diffraction spectra of YAG:2%Ce^3+^, *x*Mn^2+^ (*x* = 20%, 24% and 28%). A minor impurity phase is observed at 29.2° in the samples with *x* = 24% and 28%.


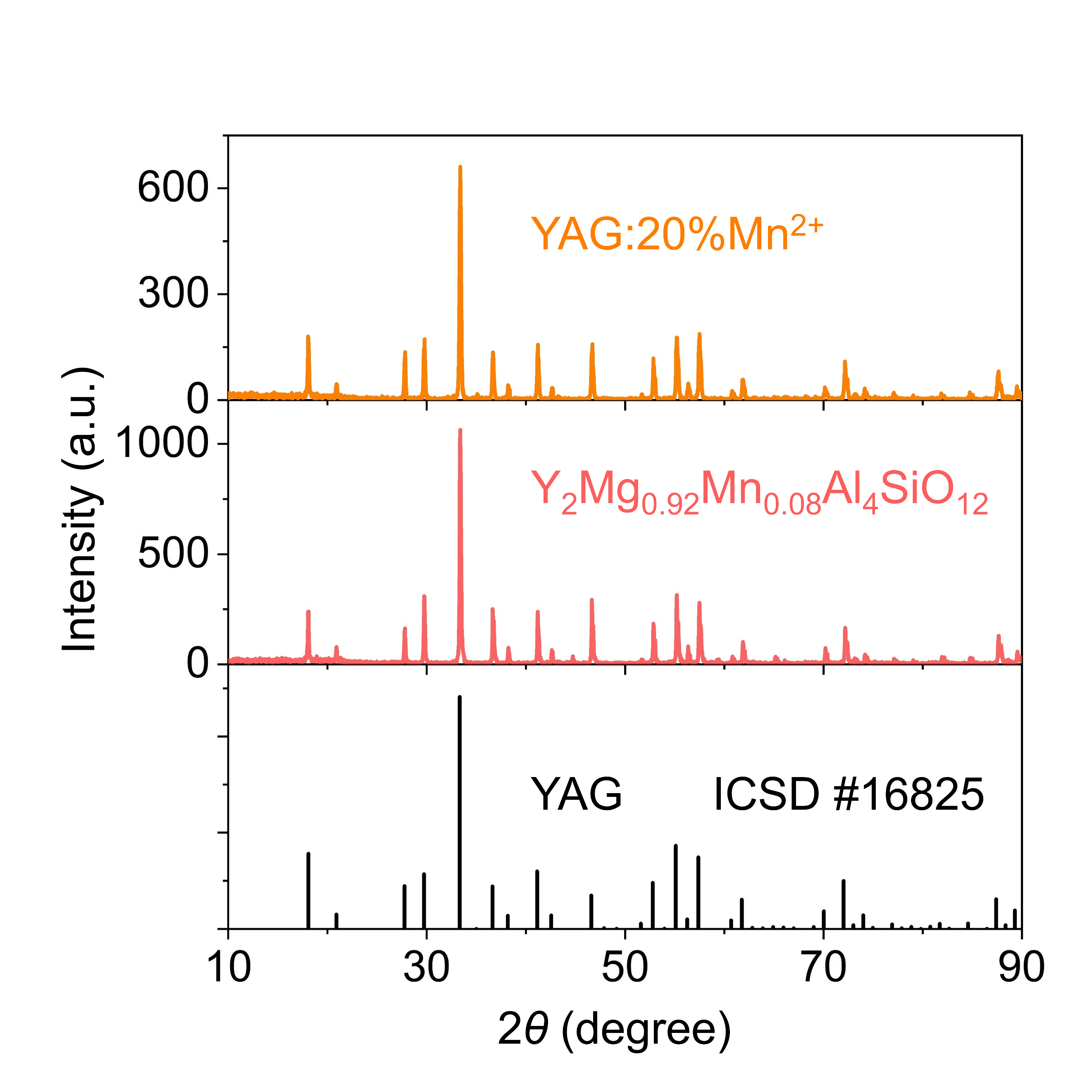


**Fig. S3** The X-ray diffraction spectra of YAG:20%Mn^2+^ and Y_2_Mg_0.92_Mn_0.08_Al_4_SiO_12_. Their structures are pure, with no evident impurities.


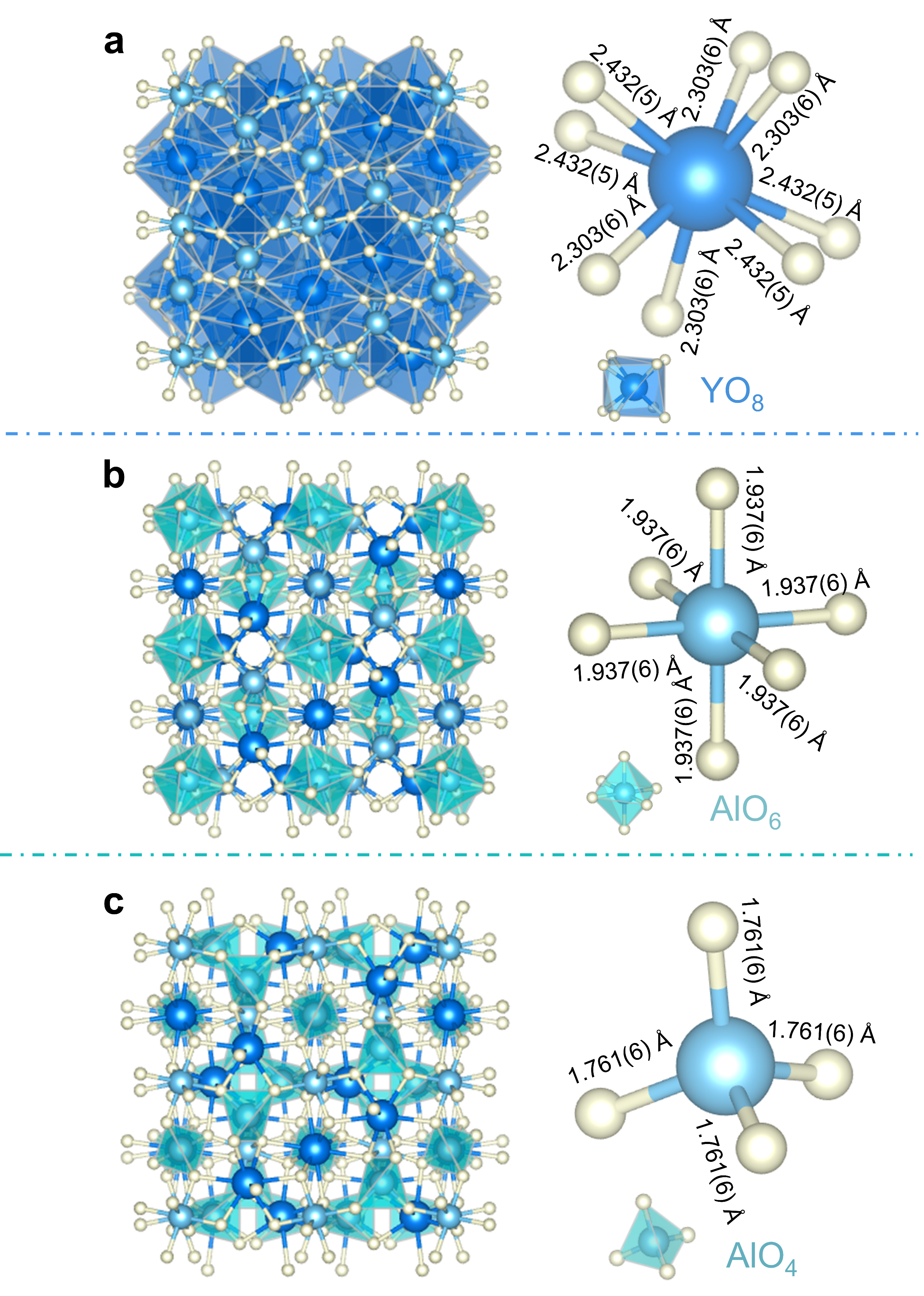


**Fig. S4** **a** Schematic structural diagram of the YAG crystal, featuring YO_8_ dodecahedrons, **b** AlO_6_ octahedrons, and **c** AlO_4_ tetrahedrons at the vertices, along with their corresponding bond lengths. Previous studies have indicated that Mn^2+^ can occupy both dodecahedral and octahedral sites within the garnet structure, thereby forming two luminescent centers responsible for red and NIR emissions. ^1^


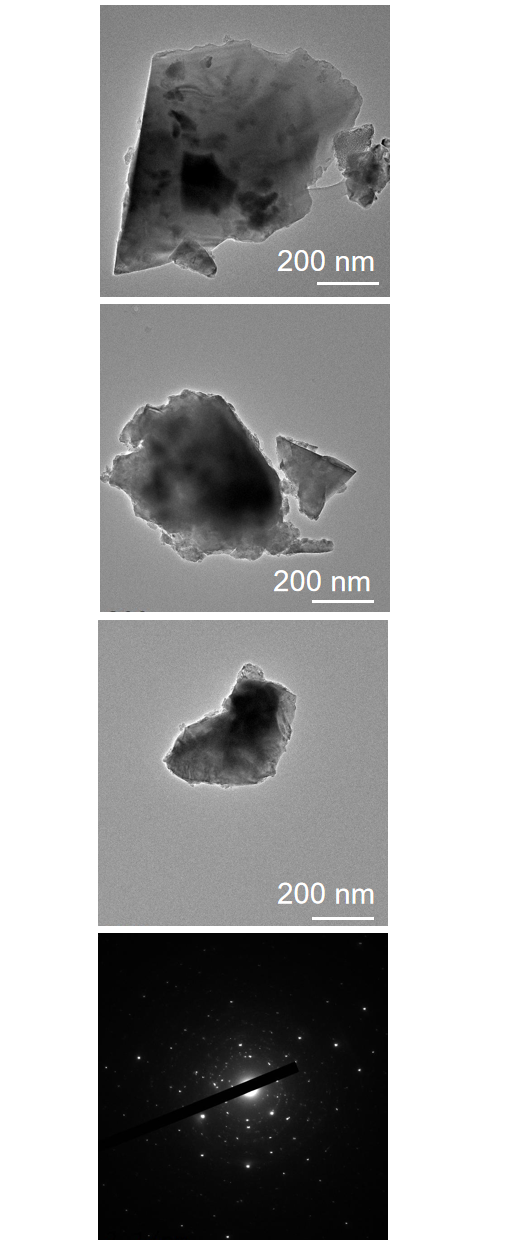


**Fig. S5** The HRTEM image and electron diffraction pattern of YAG:2%Ce^3+^, 20%Mn^2+^. YAG:2%Ce^3+^, 20%Mn^2+^ are micron-sized particles with irregular morphology and excellent crystallinity.


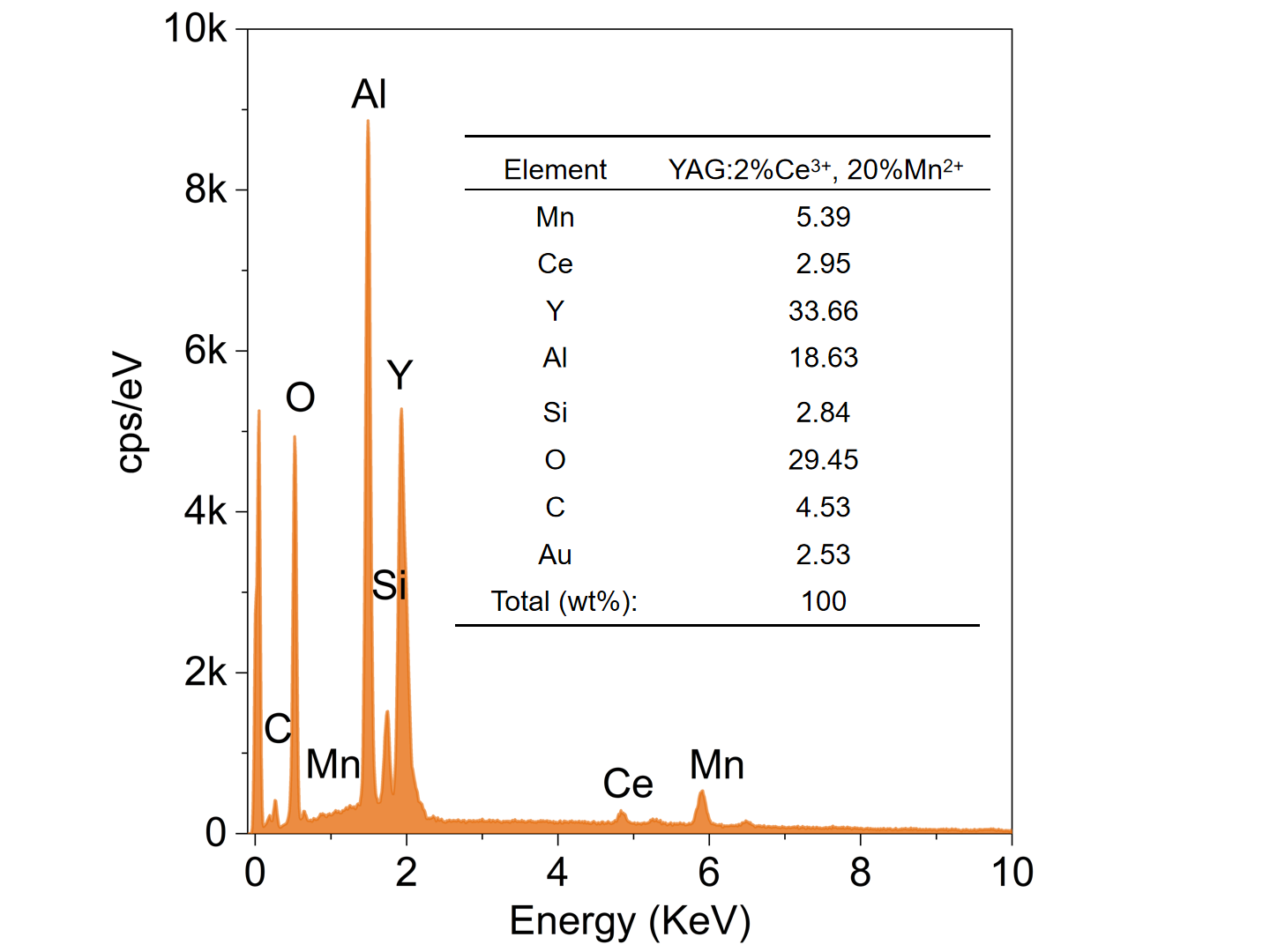


**Fig. S6** EDS spectrum of YAG:2%Ce^3+^, 20%Mn^2+^. The proportions of the monitored elements closely match those in the chemical formula.

**
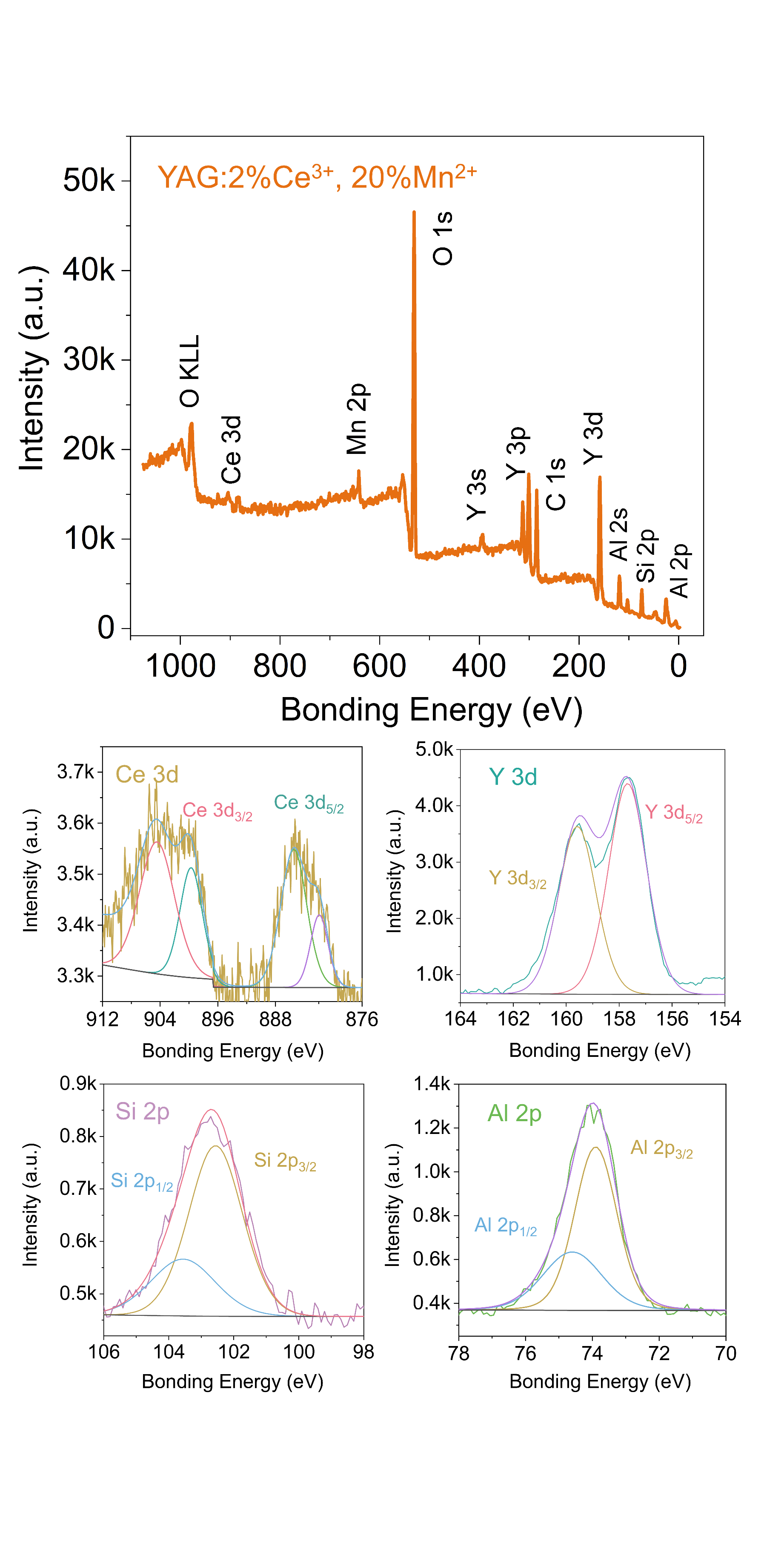
**

**Fig. S7** XPS spectrum of YAG:2%Ce^3+^, 20%Mn^2+^. The XPS results indicate that the surface of YAG:2%Ce^3+^, 20%Mn^2+^ consists of cerium (Ce), manganese (Mn), silicon (Si), yttrium (Y), aluminum (Al), and oxygen (O).


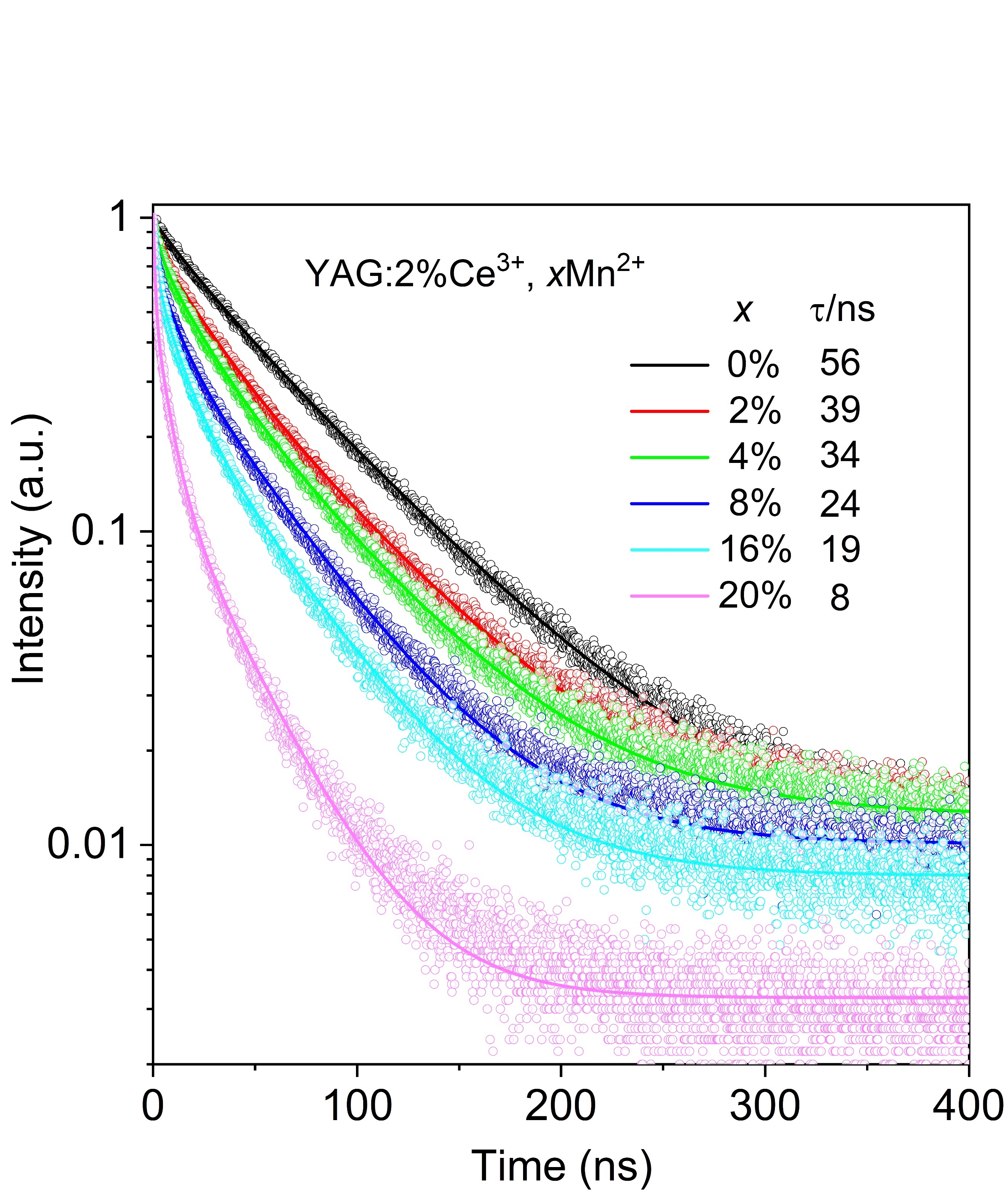


**Fig. S8** The decay curves of Ce^3+^ emission from YAG:2%Ce^3+^, *x*Mn^2+^ (*x* = 0%–20%) were monitored at 540 nm after pulse excitation at 450 nm. With the increase in Mn^2+^ doping concentration (*x*), the fluorescence lifetime of Ce^3+^ gradually decreases, indicating the presence of energy transfer from Ce^3+^ to Mn^2+^.The specific fitting equation and parameters are listed in Table S2.


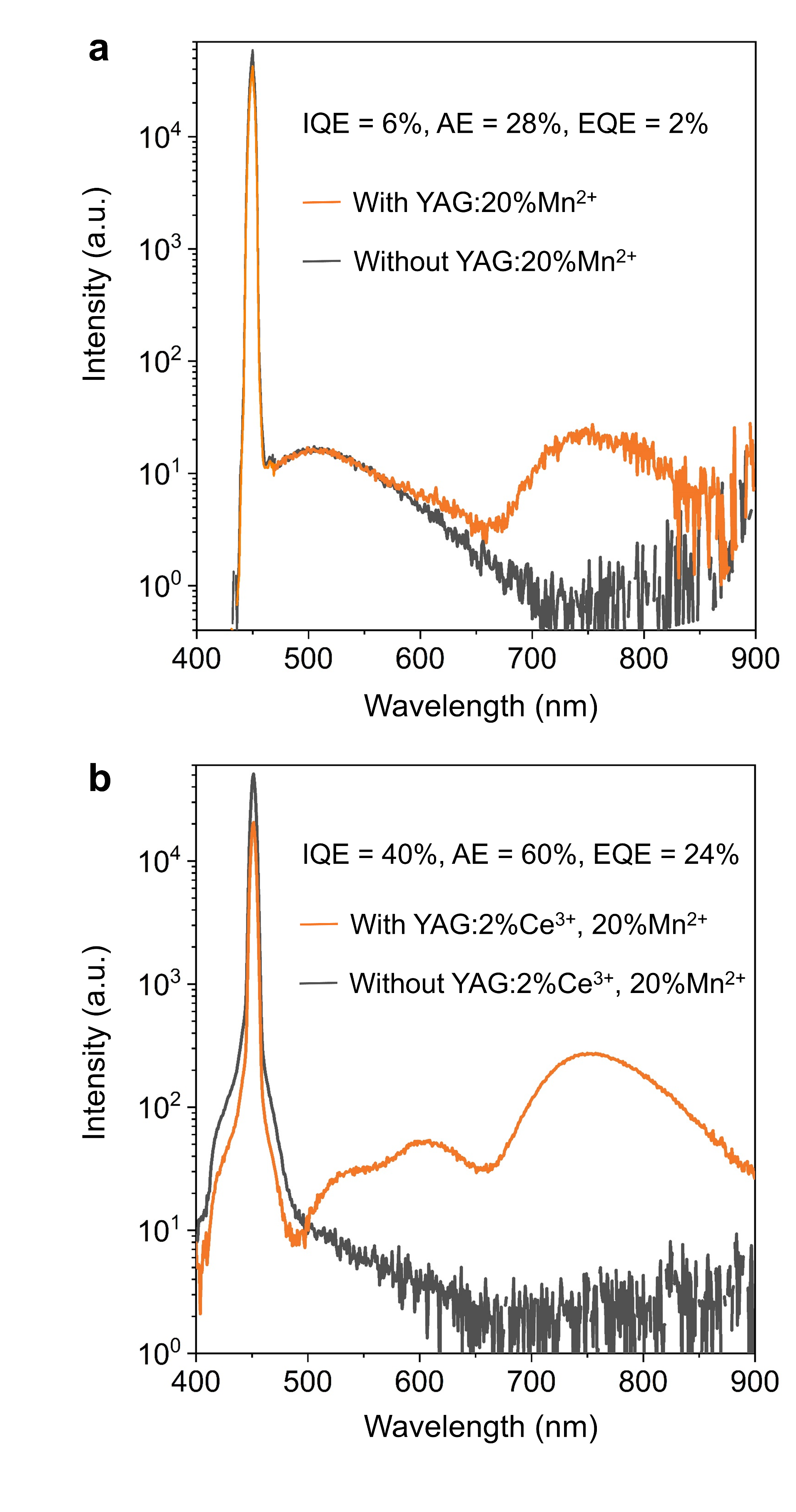


**Fig. S9** **a** The PL spectra in the integrating sphere are presented for samples with and without YAG doped with 20%Mn^2+^ and **b** YAG co-doped with 2%Ce^3+^ and 20%Mn^2+^. Additionally, the corresponding internal quantum efficiency (IQE), absorption efficiency (AE), and external quantum efficiency (EQE) are provided. When YAG is doped solely with 20%Mn^2+^, its PL spectra predominantly exhibit NIR light, yet it demonstrates a notable concentration quenching effect. However, upon the introduction of 2%Ce^3+^ as a co-dopant, significant enhancements are observed in the IQE, AE, and EQE.


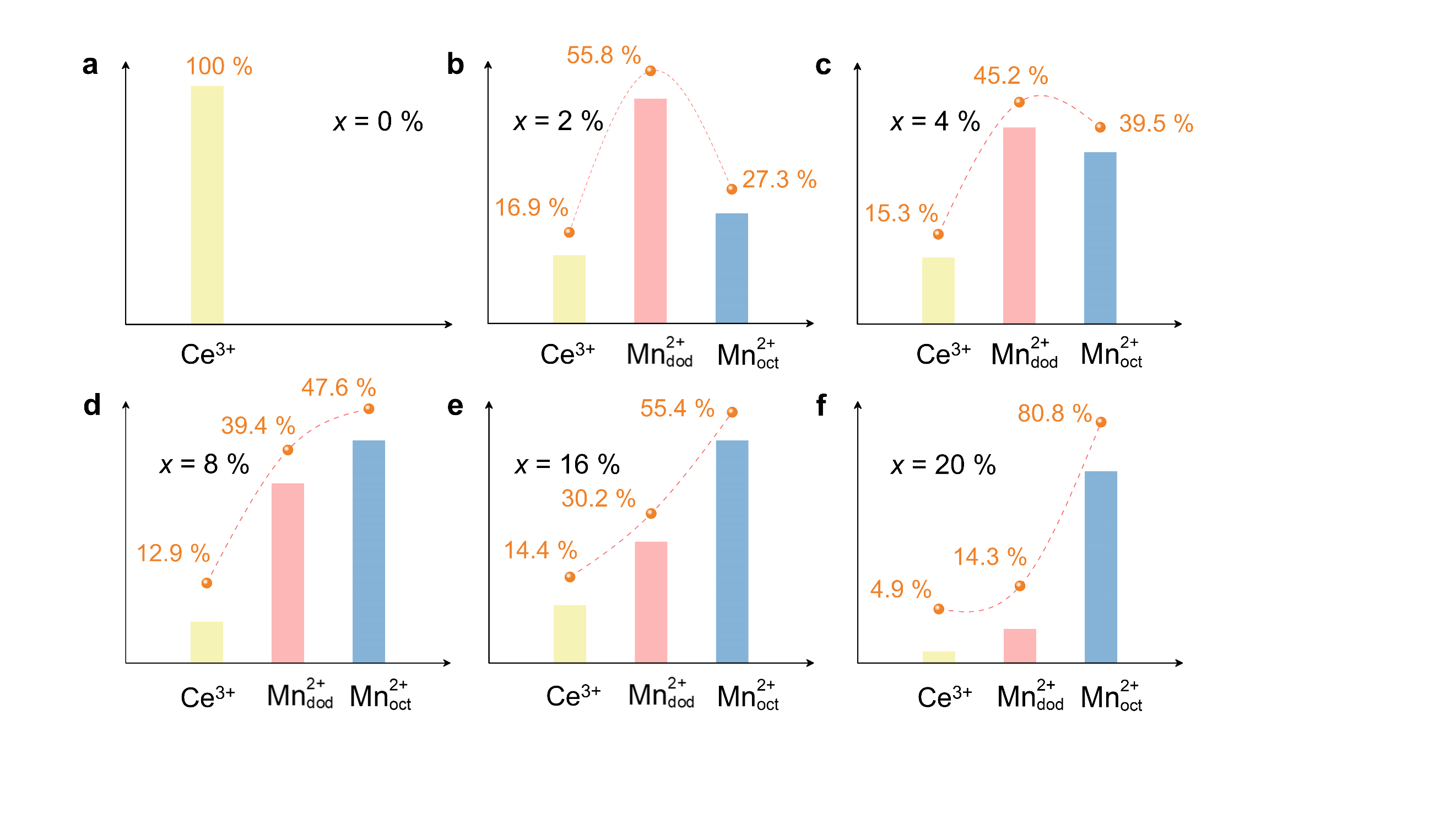


**Fig. S10** The evolution trend of the emission integral intensity pertains to Ce^3+^ (~540 nm), Mn2+ dod (~600 nm), and Mn2+ oct (~750 nm) in YAG:2%Ce^3+^, *x*Mn^2+^ (with *x* ranging from 0% to 20%) as Mn^2+^ concentration (*x*) increases. Specifically: **a** at *x* = 0%; **b** *x* = 2%; **c** *x* = 4%; **d** *x* = 8%; **e** *x* = 16%; and **f** *x* = 20%. In the subsequent sections, we demonstrate that the red emissions originate from Mn^2+^ occupying dodecahedral sites, whereas the NIR emissions arise from Mn^2+^ occupying octahedral sites. Consequently, we designate the red emission (~600 nm) centers as Mn2+ dod and the NIR emission centers as Mn2+ oct. The statistical results clearly show that Mn^2+^ primarily occupies octahedral sites to create red luminescent centers (~600 nm), after which its luminescence intensity decreases, and NIR emission (~750 nm) progressively becomes the dominant feature. At *x* = 2%, the material predominantly emits red light (~600 nm). By *x* = 8%, the proportion of NIR emission (~750 nm) surpasses that of red light (~600 nm). When *x* reaches 20%, NIR emission (~750 nm) comprises 80.8% of the total spectral integral intensity.


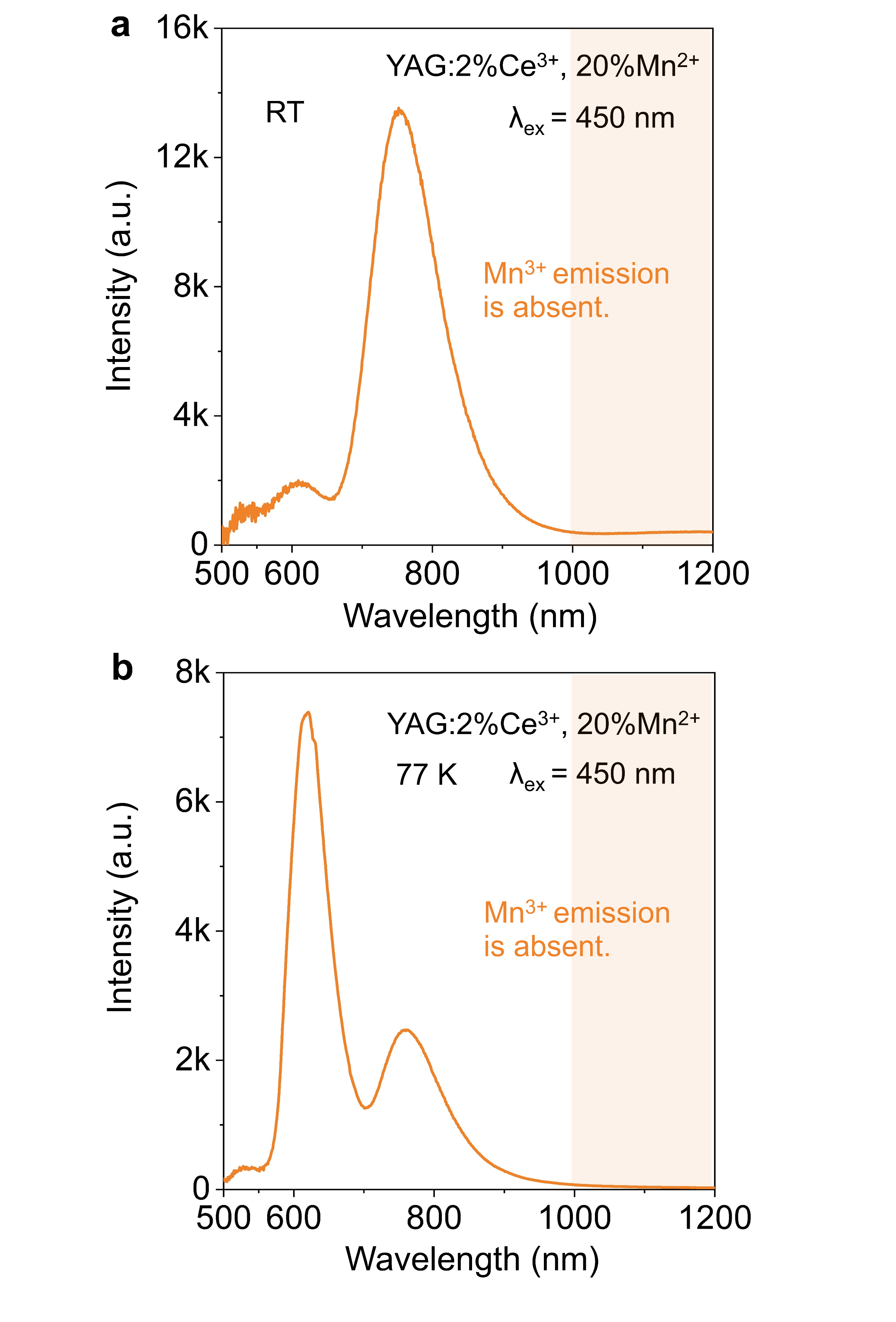


**Fig. S11** **a** PL spectra of YAG:2%Ce^3+^, 20%Mn^2+^ at RT, and **b** 77 K. By contrasting RT and 77 K spectra, we verify that the NIR emission of YAG:2%Ce^3+^, 20%Mn^2+^ does not emanate from Mn^3+^. If Mn^3+^ luminescence is present in the garnet structure, detecting the characteristic emission from the ^1^T_2_ → ^3^T_2_ transition of Mn^3+^ within the 1100 nm to 1200 nm wavelength range in 77 K spectra is feasible. The remarkable disparities between room temperature and 77 K spectra suggest potential significant alterations in energy transfer or migration within YAG:2%Ce^3+^, 20%Mn^2+^ across varying temperatures. (Supplementary note: To obtain clearer signals within the spectral range of 1000 nm to 1200 nm, the RT and 77 K spectra were acquired under distinct test conditions. Consequently, the absolute intensities presented in these two images do not serve as a comparative reference value. Future research will delve deeper into elucidating the differences in energy distribution.)

**
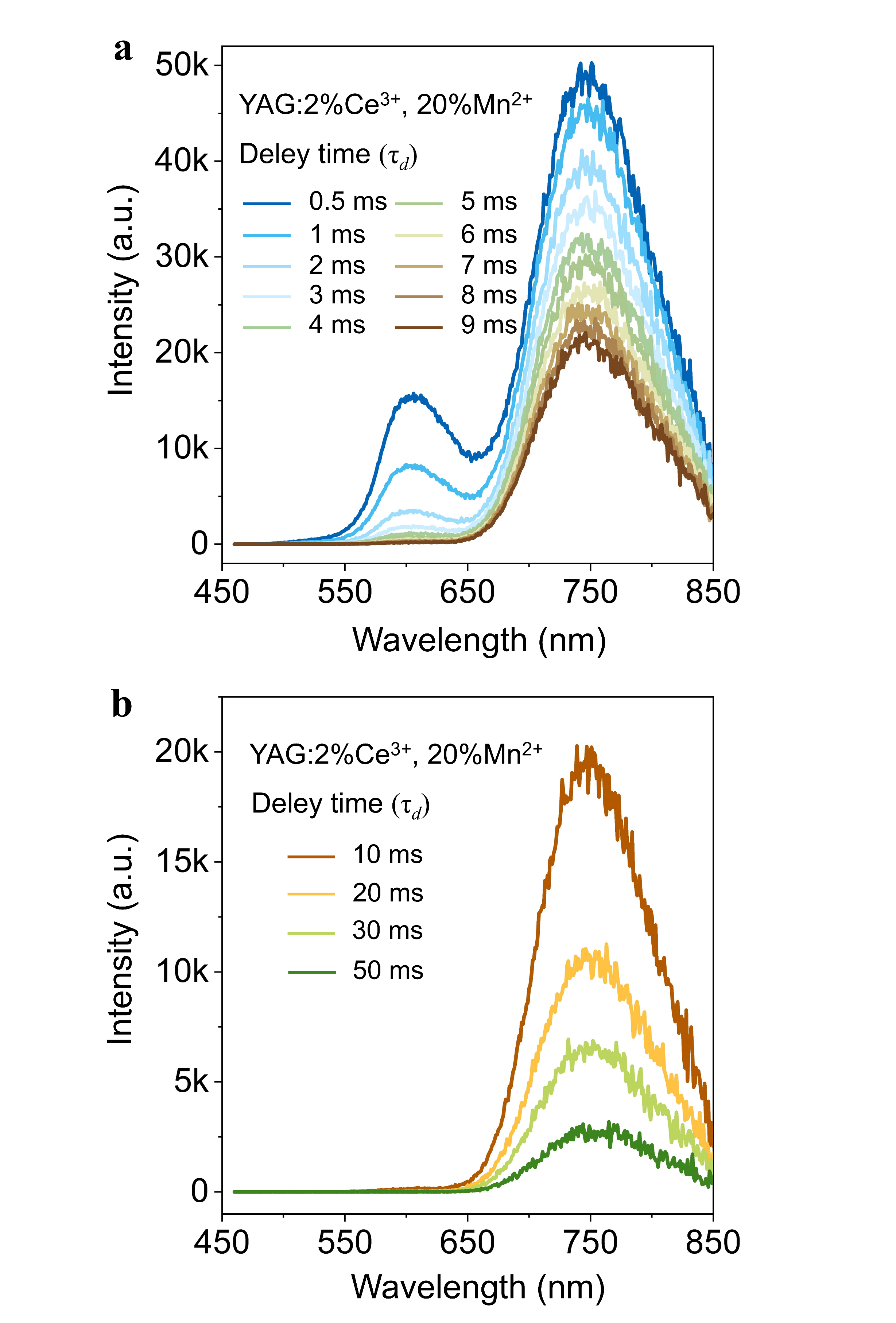
**

**Fig. S12** The time-resolved emission spectra of YAG:2%Ce^3+^, 20%Mn^2+^ were obtained with a delay time τ*_d_* ranging from 0.5 to 50 ms. **a** τ*_d_* = 0.5–9 ms；**b** τ*_d_* = 10–50 ms. Upon analyzing the time-resolved spectra of YAG:2%Ce^3+^, 20%Mn^2+^, a crucial “watershed” is observed at the 9 ms–10 ms mark. Prior to this timeframe, both red and NIR emissions coexist; however, post 10 ms, the persistence is nearly exclusive to NIR emissions, indicating the presence of two Mn^2+^ luminescence centers.


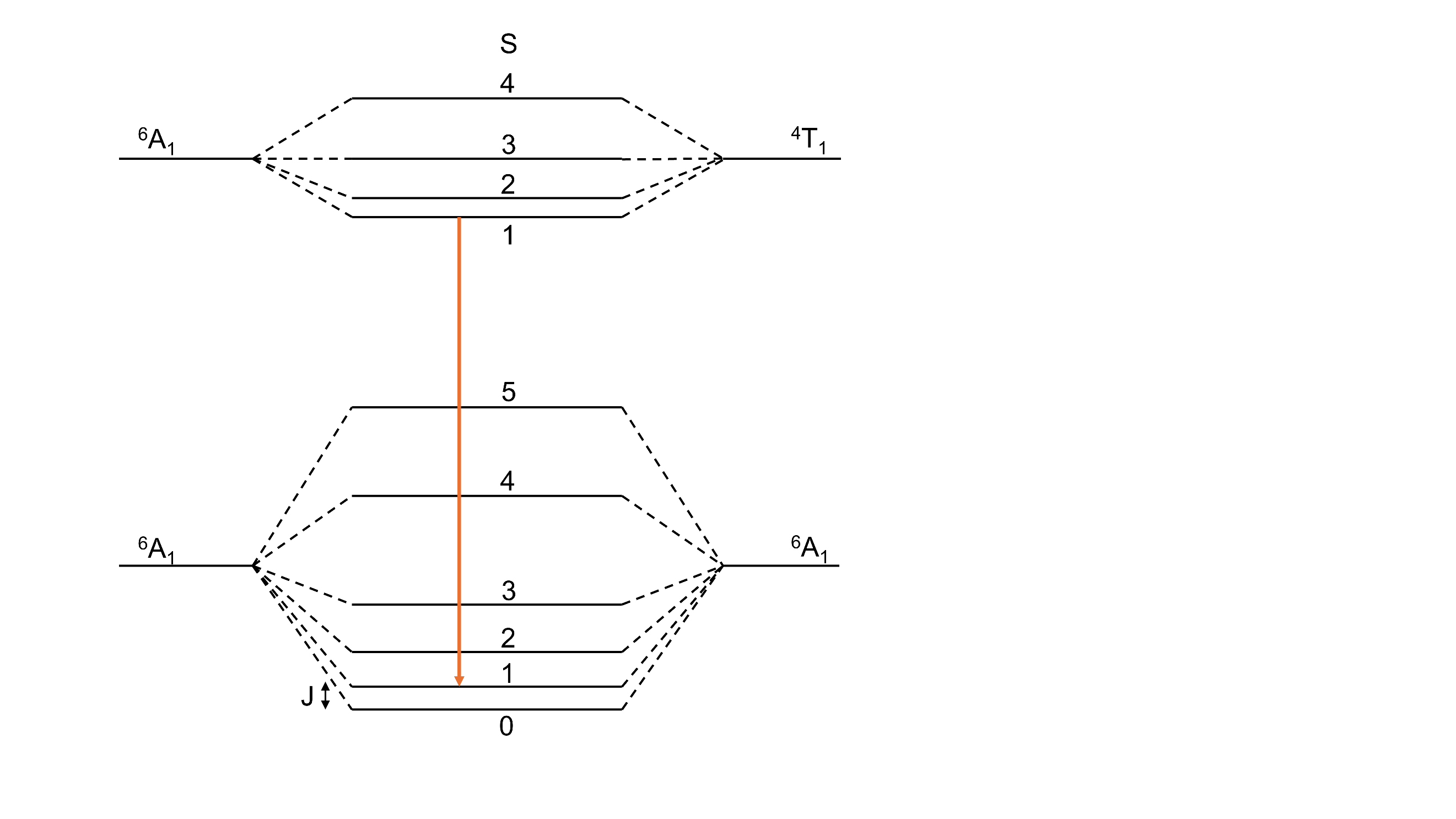


**Fig. S13** Schematic representation for the energy levels of Mn^2+^–Mn^2+^ pairs. The energy levels |^6^A_1_,^4^T_1_> and |^6^A_1_,^6^A_1_> originate from the exchange coupling for antiferromagnetic exchange-coupled Mn^2+^ pairs. The vertical purple line represents the spin allowed 1 → 1 transition (ΔS = 0). The primary effect of Mn^2+^–Mn^2+^ ion pairs is that the emission intensity of Mn^2+^ increases with increasing doping concentration, but the fluorescence lifetime significantly decreases. ^2–4^


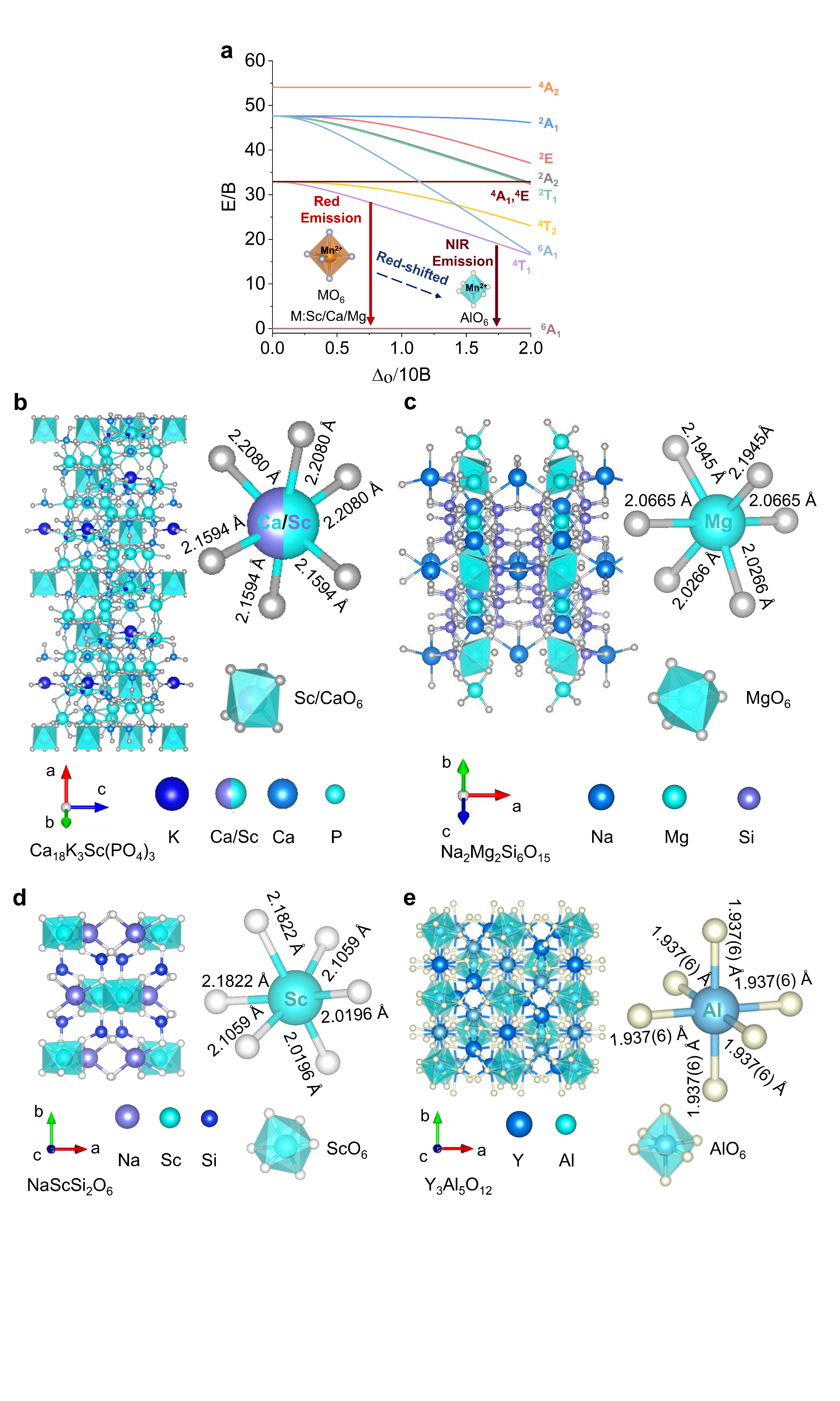


**Fig. S14** The cause of red and NIR emissions generated by Mn^2+^. **a** Schematic energy level diagrams explaining the origin of NIR emission of Mn^2+^ ions; Schematic structural diagrams of the **b** Ca_18_K_3_Sc(PO_4_)_3_, **c** Na_2_Mg_2_Si_6_O_15_, **d** NaScSi_2_O_6_, **e** Y_3_Al_5_O_12_ crystals, highlighting ScO_6_, Sc/CaO_6_, MgO_6_ and AlO_6_ octahedrons, along with their corresponding bond lengths, respectively. ^5–7^


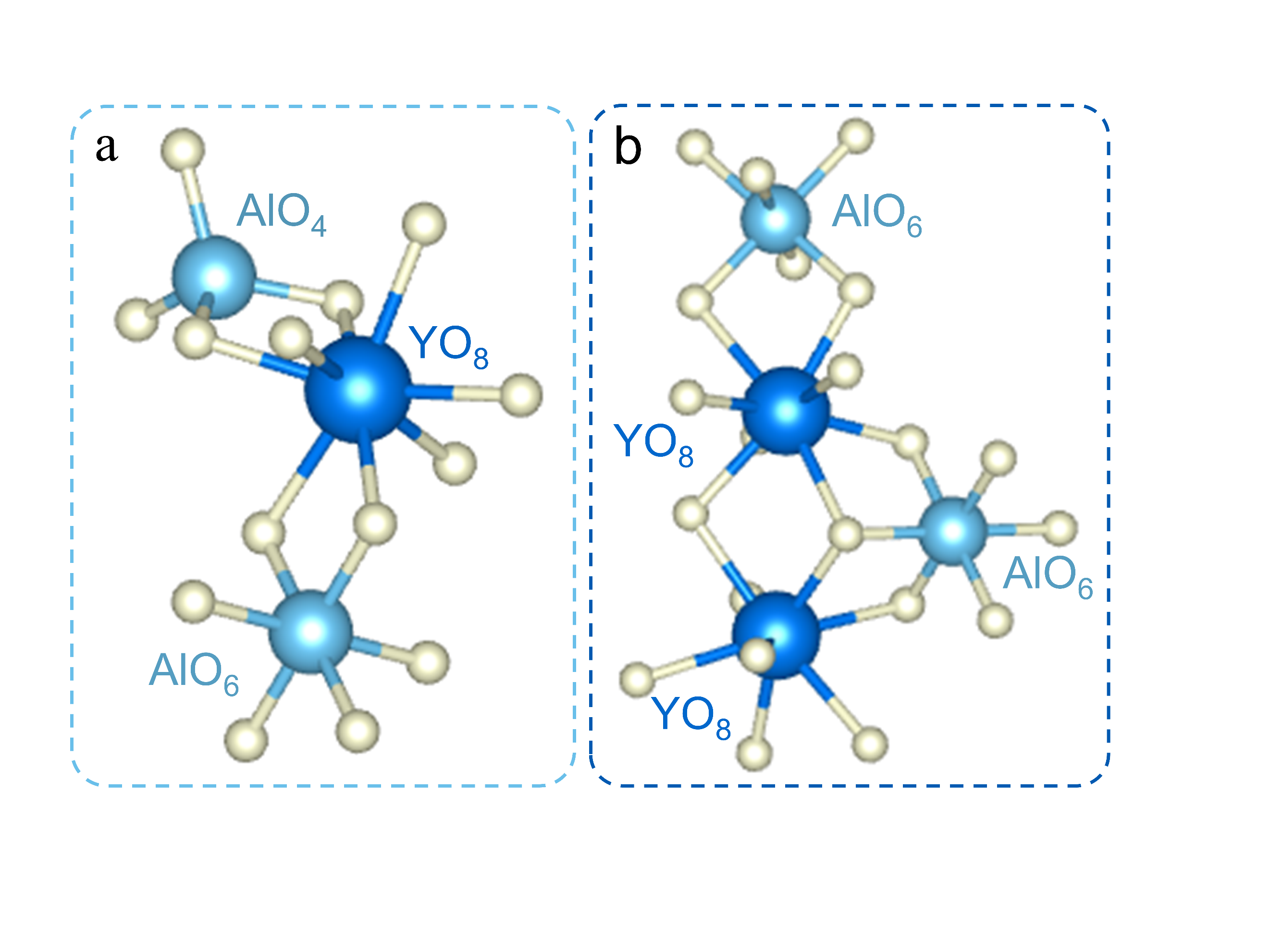


**Fig. S15** The AlO_6_ and YO_8_ sites of the YAG structure and their neighboring conditions. According to the steady-state and transient photoluminescence analysis, if there is a Mn^2+^–Mn^2+^ pair in the YAG structure, there are only two possibilities: **a** either Mn^2+^ occupies the neighboring YO_8_ and AlO_6_ sites; or **b** Mn^2+^ occupies the neighboring YO_8_ and YO_8_ sites.


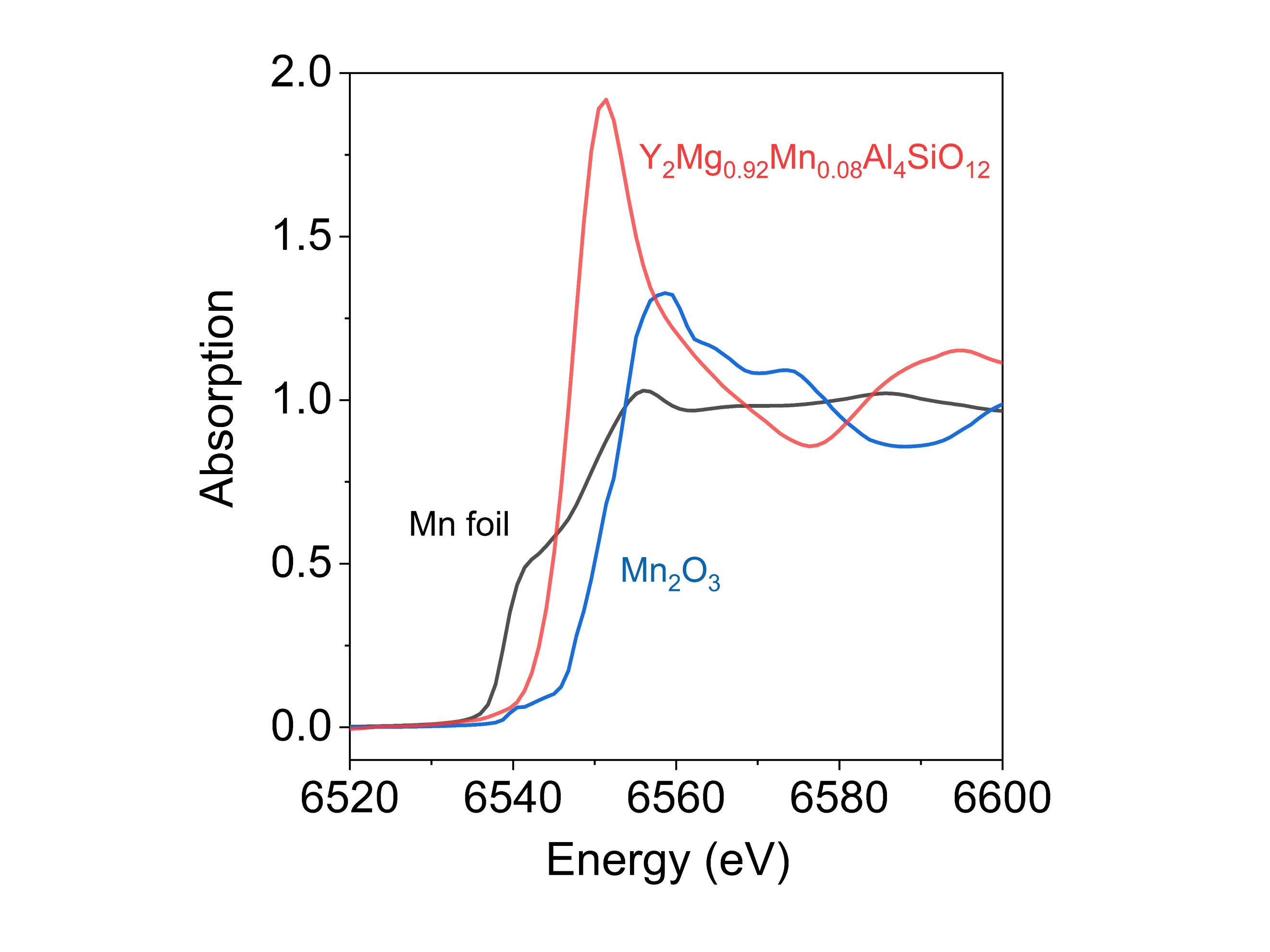


**Fig. S16** X-ray absorption near edge structure (XANES) spectra of Mn foil, Mn_2_O_3_ and Y_2_Mg_0.92_Mn_0.08_Al_4_SiO_12_. The Mn absorption edge in Y_2_MgAl_4_SiO_12_ is intermediate between the absorption edges of Mn_2_O_3_ (Mn^3+^) and Mn foil, indicating the retention of a positive divalent state for Mn (e.g., Mn^2+^) in the Y_2_MgAl_4_SiO_12_ structure.


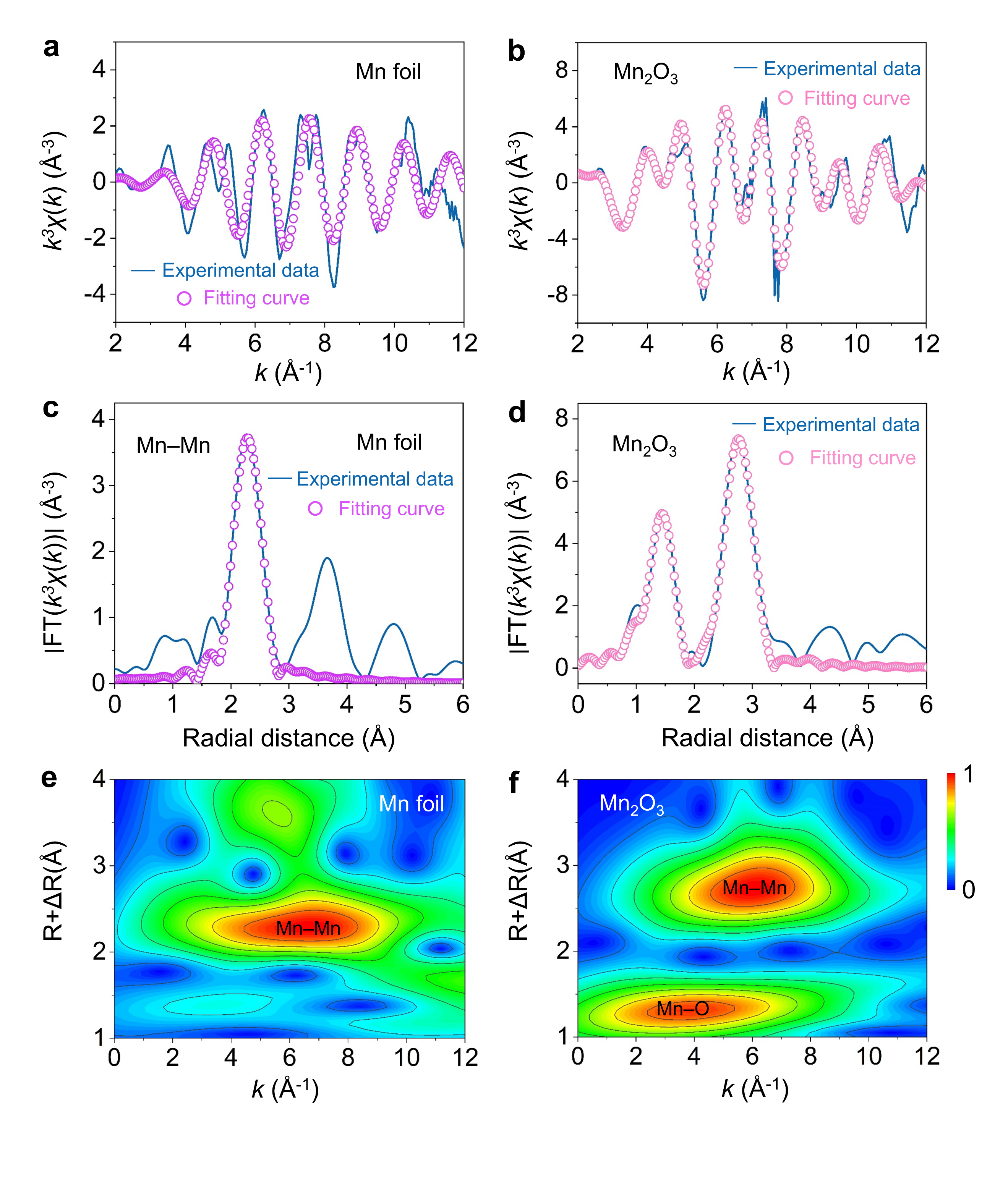


**Fig. S17** XAS characterizations of Mn foil and Mn_2_O_3_. **a–b** FT-EXAFS spectra; **c–d** Experimental and calculated EXAFS spectra χ(*k*)*k*^2^; **e–f** Wavelet transform of the *k*^3^-weighted EXAFS data.

**
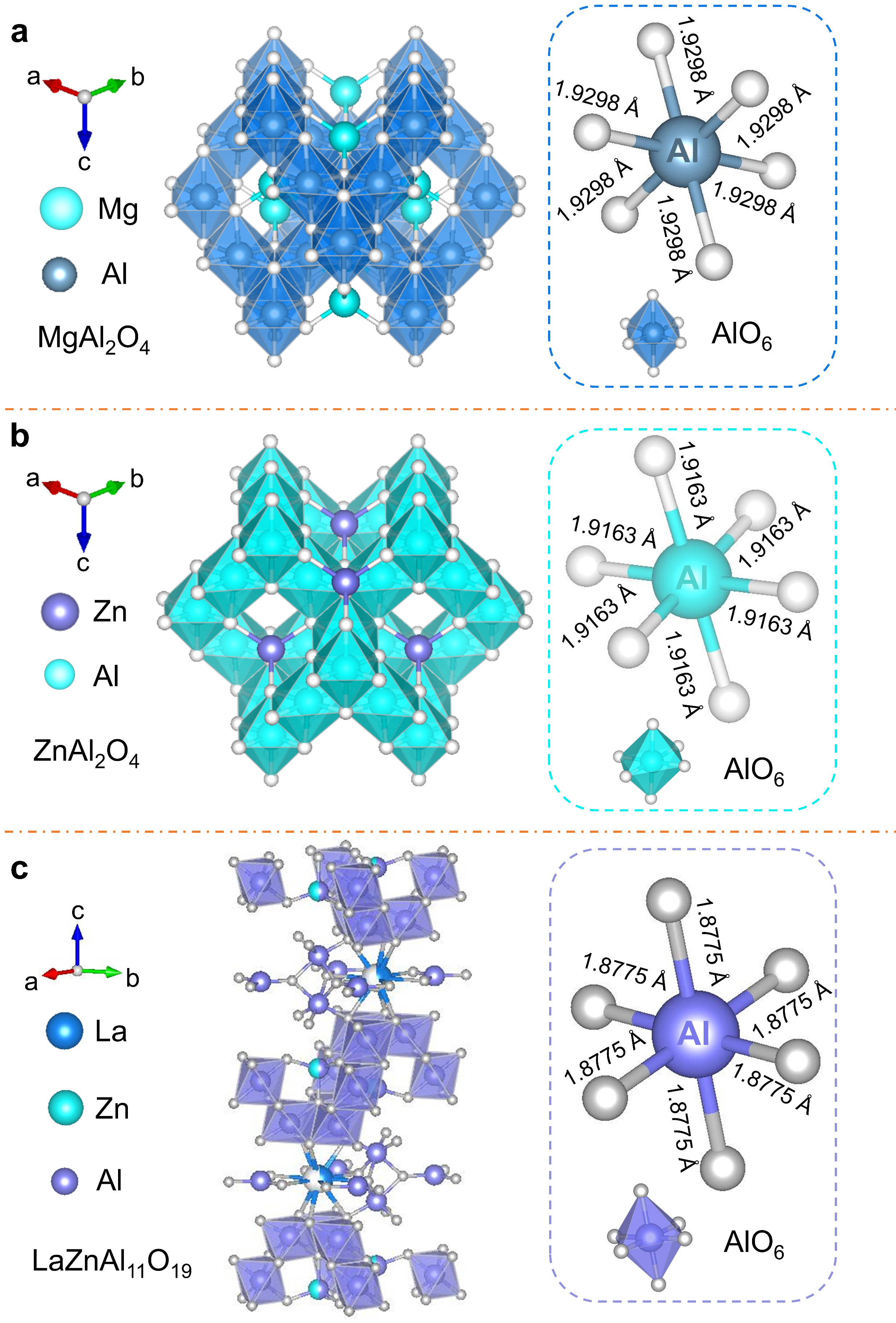
**

**Fig. S18** The schematic diagram of the crystal structure of MgAl_2_O_4_, ZnAl_2_O_4_, and LaZnAl_11_O_19_ along with the Al–O bond length within its octahedral site AlO_6_, **a–c** is presented. Previous studies have hypothesized that the NIR emissions exhibited by MgAl_2_O_4_:Mn^2+^, ZnAl_2_O_4_:Mn^2+^, and LaZnAl_11_O_19_:Mn^2+^ can be ascribed to the coupling interaction of Mn^2+^–Mn^2+^ pairs. ^8–10^


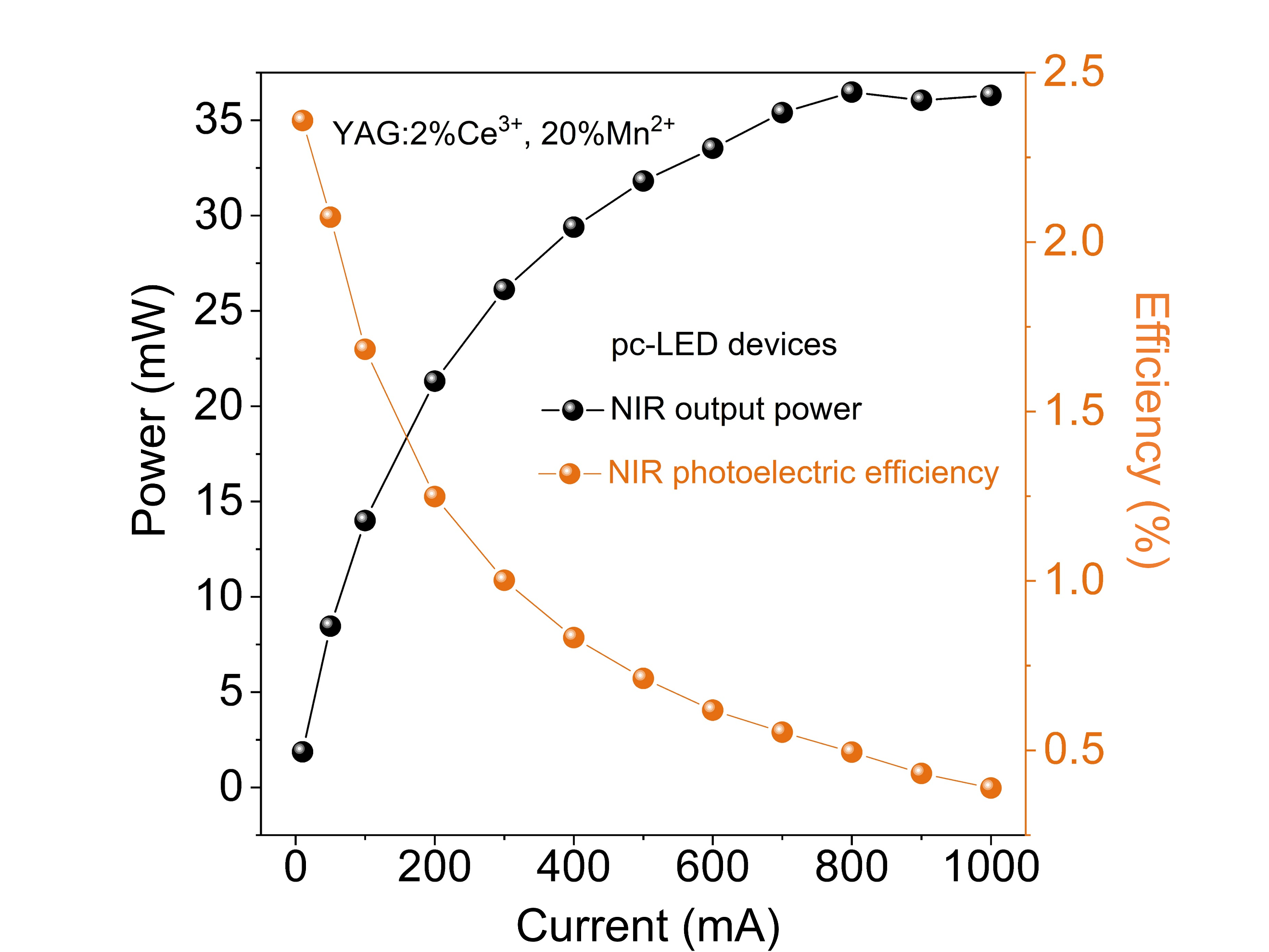


**Fig S19.** NIR output power and NIR photoelectric efficiency as a function of driving current. As the input power increases, the NIR output power also rises, reaching a “saturation state” at 800 mA. However, the photoelectric conversion efficiency gradually decreases due to the well-known “efficiency droop” phenomenon.

**
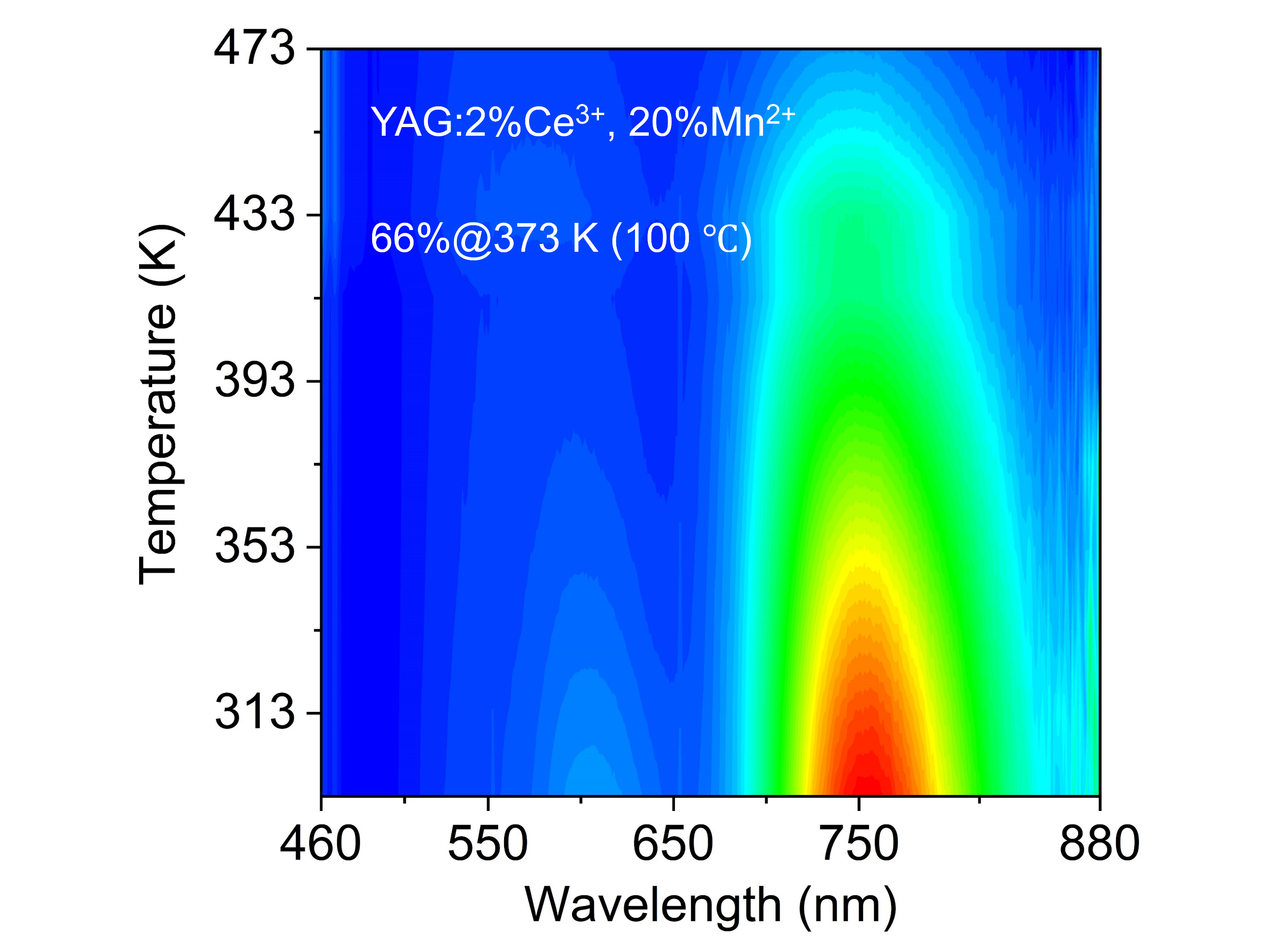
**

**Fig S20.** The temperature dependent PL spectra for YAG:2%Ce^3+^, 20%Mn^2+^. In our article, we conclusively demonstrate that red emission originates from Mn^2+^ occupying dodecahedral lattice sites (Mn2+ dod), while NIR emission arises from Mn^2+^ in octahedral lattice sites (Mn2+ oct). Notably, Mn2+ oct (~750 nm) exhibits superior luminescence thermal stability compared to Mn2+ dod (~600 nm). Research on the thermal stability of Ce^3+^/Eu^2+^ luminescence has inspired two primary mechanisms to explain the thermal stability of Mn^2+^ luminescence. The first is thermal crossover, where an increase in temperature increases the likelihood of electrons non-radiatively relaxing back to the ground state energy level through the intersection between the ^4^T_1_ excited state and the ^6^A_1_ ground state. Secondly, thermally assisted photoionization, where thermal energy elevates electrons from the excited state to the conduction band, allowing for higher mobility and subsequent capture by impurities or defects. I believe the difference in luminescent thermal stability is associated with their local environment and bandgaps. Furthermore, potential energy migration from Mn2+ dod to Mn2+ oct is also one of the potential factors. ^11–12^


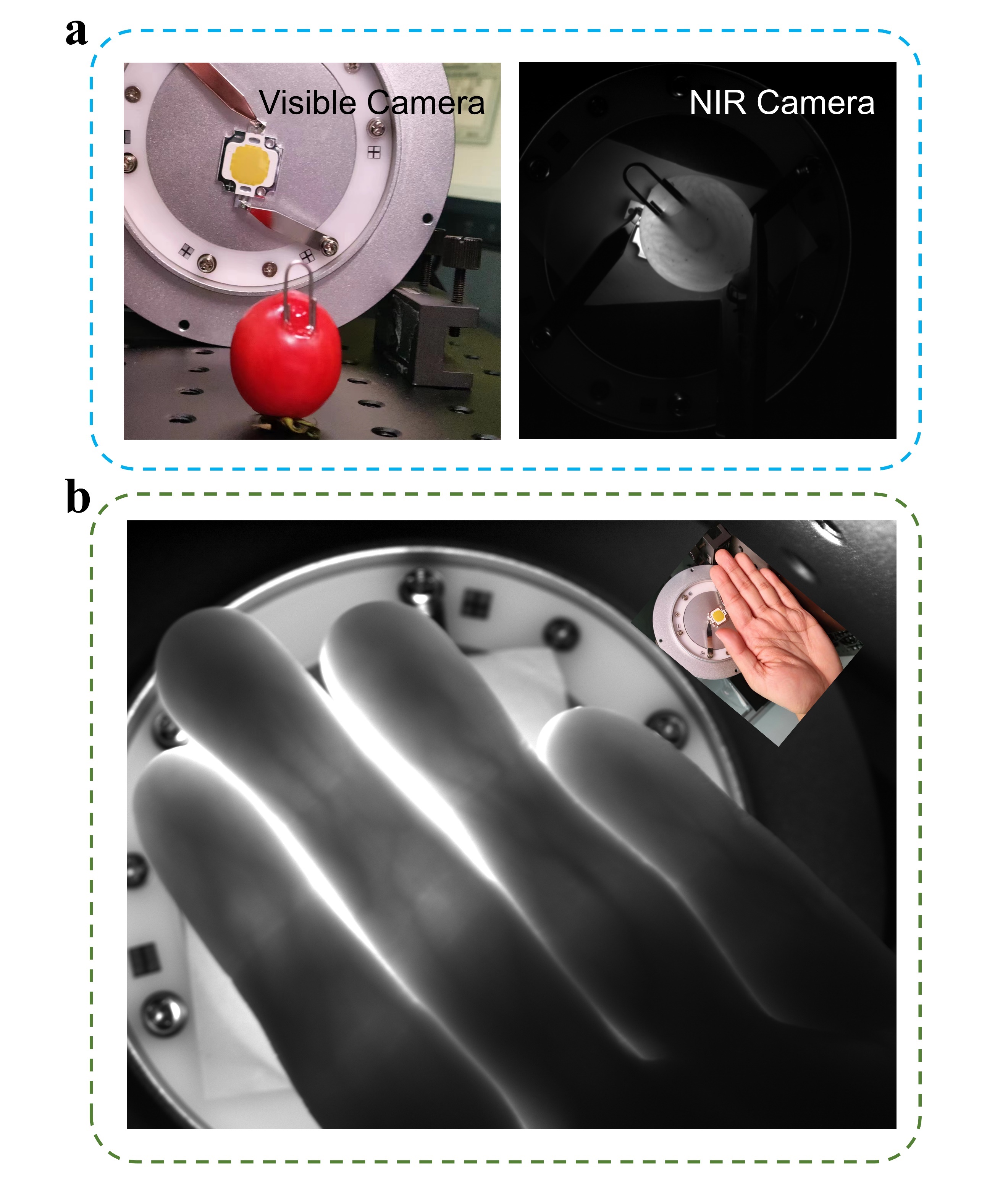


**Fig S21.** **a** Photos of the lighting pc-LEDs taken by a normal camera and a NIR camera; **b** Photo of the lighting pc-LED taken by a NIR camera and trans-illuminated photo of the finger. We illuminate the NIR pc-LEDs device and direct its light onto a cherry tomato with a paper clip inserted. The paper clip within the tomato flesh is clearly visible in the NIR camera. Similarly, we can observe the distinct distribution of blood vessels in the palm.


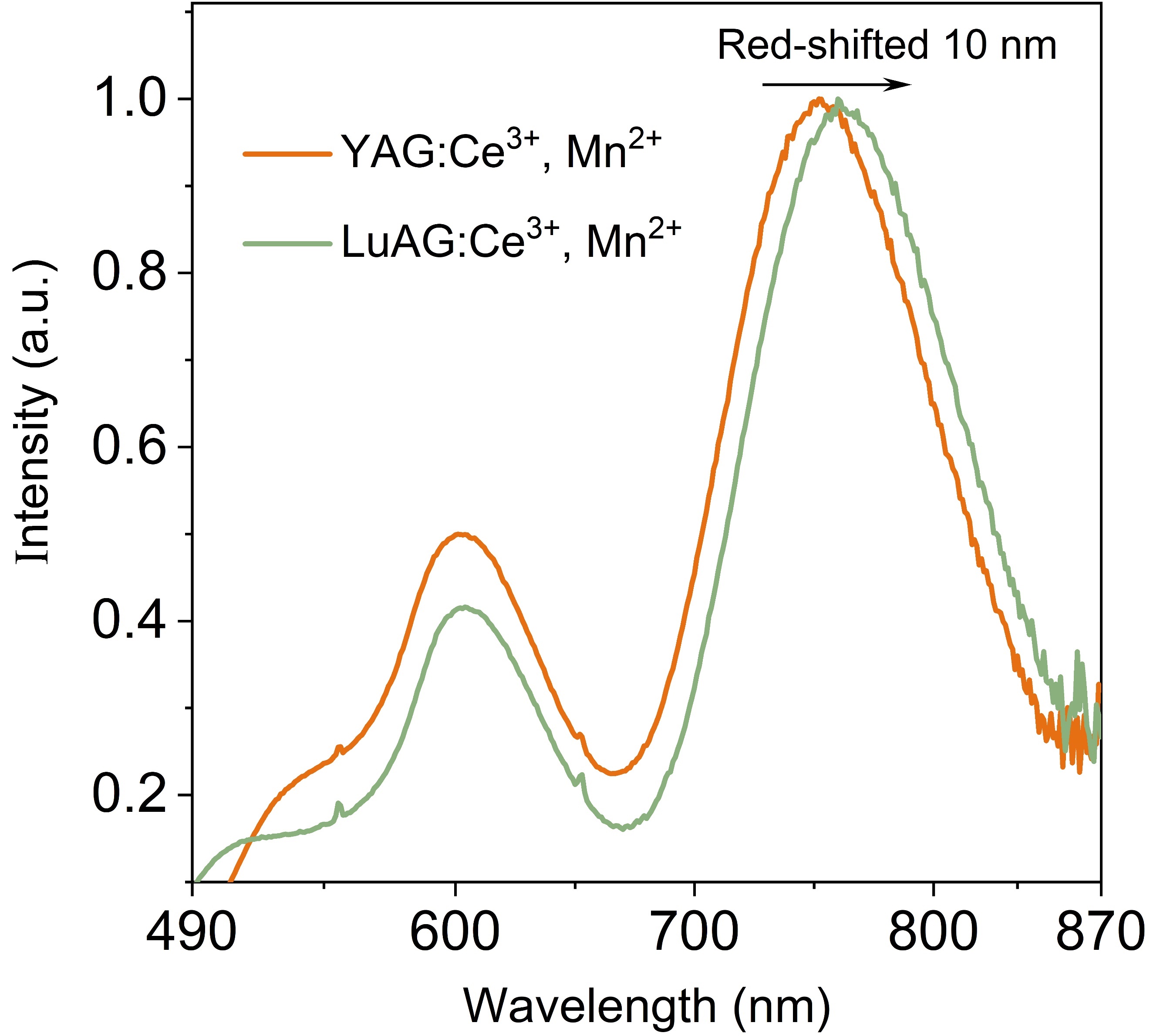


**Fig. S22** The photoluminescence spectra of YAG:Ce^3+^, Mn^2+^ and Lu_3_Al_5_O_12_:Ce^3+^, Mn^2+^ (LuAG:Ce^3+^, Mn^2+^) phosphors. The NIR emission peak of Mn^2+^ in YAG is located at approximately 750 nm, while in LuAG it is situated at around 760 nm. This indicates the potential for Mn^2+^ to effectively modulate its NIR emission.

**Supplementary Tables**

**Table S1.** The Rietveld refinement data of YAG:2%Ce^3+^, *x*Mn^2+^ (*x* = 0%–16%).

| Formula | *x* = 0% | *x* = 2% | *x* = 4% | *x* = 8% | *x* = 16% |  |
| --- | --- | --- | --- | --- | --- | --- |
| Crystal system | *Ia*$\bar{3}$*d* |  |  |  |  |  |
| Space group | 230 |  |  |  |  |  |
|  |  |  |  |  |  |  |
| *a* = *b* = *c* (Å) | 12.01020 | 12.00896 | 12.00706 | 11.99760 | 11.98528 |  |
| *α* = *β* = *γ* (°) |  |  |  |  |  |  |
| Cell Volume | 1732.410 | 1731.873 | 1731.052 | 1726.964 | 1721.651 |  |
| R_p_(%) | 1.46 | 1.42 | 1.41 | 1.37 | 1.41 |  |
| R_wp_(%) | 1.93 | 1.85 | 1.83 | 1.75 | 1.82 |  |
| χ^2^ | 2.12 | 1.83 | 1.78 | 1.68 | 1.73 |  |

**Table S2.** The lifetimes of YAG:2%Ce^3+^, *x*Mn^2+^ (*x* = 0%–20%) were monitored at 540 nm after pulse excitation at 450 nm, along with their respective fitting functions and parameters.

| Formula | *x* = 0% | *x* = 2% | *x* = 4% | *x* = 8% | *x* = 16% | *x* = 20% |
| --- | --- | --- | --- | --- | --- | --- |
| Fitting  function | y = y_0_+A_1_exp(-*x*/t_1_) + A_2_exp(*x*/t_2_)+ A_3_exp(*x*/t_3_) | | | | | |
| Fitting  parameter | A_1_ = 0.0718  A_2_ = 0.8304  A_3_ = 0.0786 | A_1_ = 0.1186  A_2_ = 0.2784  A_3_ = 0.5507 | A_1_ = 0.1662  A_2_ = 0.3264  A_3_ = 0.4740 | A_1_ = 0.2241  A_2_ = 0.3142  A_3_ = 0.4394 | A_1_ = 0.3039  A_2_ = 0.3534  A_3_ = 0.3222 | A_1_ = 0.5054  A_2_ = 0.3555  A_3_ = 0.1611 |
| Component  lifetime (ns) | τ_1_ = 11.499  τ_2_ = 55.745  τ_3_ = 129.02 | τ_1_ = 2.7988  τ_2_ = 21.894  τ_3_ = 59.190 | τ_1_ = 3.2270  τ_2_ = 19.903  τ_3_ = 56.254 | τ_1_ = 0.9039  τ_2_ = 10.067  τ_3_ = 46.498 | τ_1_ = 1.5835  τ_2_ = 11.268  τ_3_ = 44.232 | τ_1_ = 0.9757  τ_2_ = 7.0474  τ_3_ = 31.960 |
| R^2^ | 0.9994 | 0.9992 | 0.9992 | 0.9997 | 0.9989 | 0.9988 |
| Ce^3+^ lifetime | 57 ns | 39 ns | 34 ns | 24 ns | 19 ns | 8 ns |

**Table S3.** The lifetimes of YAG:2%Ce^3+^, *x*Mn^2+^ (*x* = 0%–16%) were monitored at 600 nm after pulse excitation at 450 nm, along with their respective fitting functions and parameters.

| Formula | *x* = 2% | *x* = 4% | *x* = 8% | *x* = 16% | *x* = 20% |
| --- | --- | --- | --- | --- | --- |
| Fitting  function | y = y_0_+A_1_exp(-*x*/t_1_) + A_2_exp(*x*/t_2_)+ A_3_exp(*x*/t_3_) | | | | |
| Fitting  parameter | A_1_ = 0.4657  A_2_ = 0.3939  A_3_ = 0.0242 | A_1_ = 0.4066  A_2_ = 0.3551  A_3_ = 0.2556 | A_1_ = 0.4852  A_2_ = 0.2885  A_3_ = 0.2299 | A_1_ = 0.3266  A_2_ = 0.5048  A_3_ = 0.1673 | A_1_ = 0.3855  A_2_ = 0.4352  A_3_ = 0.1743 |
| Component  lifetime (ms) | τ_1_ = 0.7709  τ_2_ = 2.2307  τ_3_ = 6.0986 | τ_1_ = 0.0435  τ_2_ = 0.8248  τ_3_ = 2.7241 | τ_1_ = 0.0373  τ_2_ = 0.7093  τ_3_ = 2.6002 | τ_1_ = 0.0475  τ_2_ = 0.5925  τ_3_ = 2.4179 | τ_1_ = 0.0381  τ_2_ = 0.5687  τ_3_ = 2.4970 |
| R^2^ | 0.9978 | 0.9994 | 0.9997 | 0.9998 | 0.9997 |
| Mn2+ dod lifetime | 1.38 ms | 1.01 ms | 0.82 ms | 0.72 ms | 0.69 ms |

**Table S4.** The lifetimes of YAG:2%Ce^3+^, *x*Mn^2+^ (*x* = 0%–20%) were monitored at 750 nm after pulse excitation at 450 nm, along with their respective fitting functions and parameters.

| Formula | *x* = 2% | *x* = 4% | *x* = 8% | *x* = 16% | *x* = 20% |
| --- | --- | --- | --- | --- | --- |
| Fitting  function | y = y_0_+A_1_exp(-*x*/t_1_) + A_2_exp(*x*/t_2_)+ A_3_exp(*x*/t_3_) | | | | |
| Fitting  parameter | A_1_ = 0.3645  A_2_ = 0.2834  A_3_ = 0.2194 | A_1_ = 0.3713  A_2_ = 0.3089  A_3_ = 0.2981 | A_1_ = 0.2627  A_2_ = 0.3121  A_3_ = 0.3745 | A_1_ = 0.2042  A_2_ = 0.4042  A_3_ = 0.3774 | A_1_ = 0.1671  A_2_ = 0.4292  A_3_ = 0.3780 |
| Component  lifetime (ms) | τ_1_ = 0.4751  τ_2_ = 4.9082  τ_3_ = 29.123 | τ_1_ = 0.5977  τ_2_ = 5.8503  τ_3_ = 29.660 | τ_1_ = 0.5255  τ_2_ = 5.3101  τ_3_ = 27.424 | τ_1_ = 0.5908  τ_2_ = 6.0915  τ_3_ = 26.345 | τ_1_ = 1.0702  τ_2_ = 7.1256  τ_3_ = 26.869 |
| R^2^ | 0.9975 | 0.9981 | 0.9982 | 0.9990 | 0.9993 |
| Mn2+ oct lifetime | 8.0 ms | 10.9 ms | 12.1 ms | 12.5 ms | 13.4 ms |

**Table S5.** Experimental and calculated transition energies of Mn^2+^ in YAG hosts.

|  | | | **Absorption (eV)** | | | **Emission (eV)** | | |
| --- | --- | --- | --- | --- | --- | --- | --- | --- |
| **Sites** | **E_ZPL_** | **ground** | **excite** | **excitation** | **ground** | **excite** | **emission** | **stokes shift** |
| Mn2+ oct | 2.190 | 1305.535 | 1303.209 | 2.326 | 1304.918 | 1303.438 | 1.480 | 0.229 |
| Mn2+ oct– Mn2+ dod | 2.200 | 1302.538 | 1300.113 | 2.426 | 1301.895 | 1300.330 | 1.565 | 0.217 |
| Mn2+ dod | 2.380 | 1299.862 | 1297.170 | 2.692 | 1299.245 | 1297.374 | 1.871 | 0.204 |
| Mn2+ dod– Mn2+ dod | 2.400 | 1297.739 | 1294.998 | 2.741 | 1297.122 | -1295.212 | 1.910 | 0.214 |

**Table S6.** EXAFS fitting parameters at the Mn K-edge for Mn foil (Ѕ_0_^2^ = 0.718)

| Sample | Mn foil |  |
| --- | --- | --- |
| Shell | Mn–Mn |  |
| CN^a^ | 6* |  |
| R(Å)^b^ | 2.643±0.017 |  |
| σ^2^(Å^2^)^c^ | 0.0065±0.0022 |  |
| ΔE_0_(eV)^d^ | 4.7±2.9 |  |
| R factor | 0.0166 |  |

^a^CN, coordination number; ^b^R, the distance to the neighboring atom; ^c^σ^2^, the Mean Square Relative Displacement (MSRD); ^d^ΔE_0_, inner potential correction; R factor indicates the goodness of the fit. S_0_^2^ was fixed to 0.718, according to the experimental EXAFS fit of Mn foil by fixing CN as the known crystallographic value. * This value was fixed during EXAFS fitting, based on the known structure of Mn. Fitting range: 3.0 ≤ k(Å) ≤ 11.2 and 1.0 ≤ R(Å) ≤ 3.0 (Mn foil). A reasonable range of EXAFS fitting parameters: 0.700 < Ѕ_0_^2^ < 1.000; CN > 0; σ^2^ > 0 Å^2^; |ΔE_0_| < 10 eV; R factor < 0.02.

**Supplementary Formula**

**Equation S1:** ^13–16^

$$H=\beta B_{0}\cdot g\cdot S+D\left[S_{Z}^{2}-\left(1/3\right)S\left(S+1\right)\right]+E\left(S_{X}^{2}-S_{Y}^{2}\right)+I\cdot A\cdot S$$

In Equation 1, B_0_ is the external magnetic field, β is Bohr magnetron, g is a g tensor, D is zero field splitting parameter, E is the orthorhombic distortion term, I = 5/2 is the nuclear spin of ^55^Mn and is the hyperfine interaction tensor. The electron configuration of Mn^2+^ ion is 3d^5^ (S state). In the case of d^5^ metal ions, it is known that the axial distortion of octahedral symmetry gives rise to three Kramers doublets |±5/2>, |±3/2> and |±1/2>. The resonance at g ≈ 2.0 is due to Mn^2+^ ions in an environment close to an octahedral symmetry and is known to arise from the transition between the energy levels of the lower doublet |±1/2>.

**Equation S2:** ^17–18^

The lifetime decay curves and average lifetime have been analyzed by the following equation:

$$I(t)=I_{0}+A_{1}\exp(-t/\tau_{1})+A_{2}\exp(-t/\tau_{2})+A_{3}\exp(-t/\tau_{3})$$

$$\tau=\int_{0}^{\infty} tI(t)dt/\int_{0}^{\infty} I(t)dt$$

In Equation 2, I and I_0_ are the luminescence intensities at times t and 0. A_1_, A_2_ and A_3_ are fitting constants, and τ_1_, τ_2_ and τ_3_ are the short and long lifetimes for exponential components, respectively.

**Equation S3:** ^19–20^

$$\eta_{T}=1-\frac{\tau}{\tau_{0}}$$

In Equation 3, τ and τ_0_ represent the lifetimes of Ce^3+^ doped and undoped with *x*Mn^2+^ (*x* = 2%–20%) in YAG, respectively. Using Equation 1, the energy transfer efficiency (*η*_T_) from Ce^3+^ to Mn^2+^ is determined to be 86% at *x* = 20%.

**Equation S4:** ^21^

$$\eta_{IQE}=\frac{\int L_{S}}{\int E_{R}-\int E_{S}}$$

$$\varepsilon_{AE}=\frac{\int E_{R}-\int E_{S}}{\int E_{R}}$$

$$\eta_{EQE}=\eta_{IQE}\times\varepsilon_{AE}$$

In Equation 4, Ls refers to integration of the emission spectrum of YAG:2%Ce^3+^, 20%Mn^2+^, and E_R_ and E_S_ refer to spectra of excitation light with and without the samples in the integrated sphere, respectively.

**Equation S5:** ^2–4^

$$H_{AB}=-J\left(S_{A}S_{B}\right)+j\left(S_{A}S_{B}\right)^{2}$$

$$E\left(S\right)=-J\left[S(S+1)-S_{A}(S_{A}+1)-S_{B}\left(S_{B}+1\right)\right]$$

In Equation 3, the Heisenberg Hamiltonian, H_AB_, describing the spin interaction between two transition metal ions A and B. The energy is dependent on the total spin, S, and on the exchange coupling strength parameter J. The S can take all integer values between the sum and difference of the values of S_A_ and S_B_. States with different values for S are at different energies. The sign of J is determined by the type of magnetic interaction, J > 0 for a ferromagnetic and J < 0 for an antiferromagnetic interaction. For Mn^2+^ (3d^5^), when both ions are in the ground state, ^6^A_1_ (S = 5/2), both spins have the same value: S_A_ = S_B_ = 5/2. The values of S for the exchange coupled pair can be: (S_A_ + S_B_), ..., (S_A_ – S_B_), so S = 5, 4, 3, 2, 1, 0. When one of the Mn^2+^ ions is in the first excited state, ^4^T_1_ (S = 5/2), and the other ion is in the ground state, ^6^A_1_ (S = 3/2), the total S value can be S = 4, 3, 2, and 1. Transitions where ΔS = 0 are spin allowed.

**Equation S6:** ^22–23^

$$D_{q}=\frac{ze^{2}r^{4}}{6R^{5}}$$

In Equation 4, D_q_ represents the measure of crystal field strength, R denotes the distance between the central ion and its ligands, z indicates the charge or valence of the coordinating anions, e signifies the charge of an electron, and r stands for the radius of the d wave function. For an octahedral coordination, the crystal field splitting is 10D_q._ As the bond length or polyhedral volume increases, the crystal field splitting decreases. The crystal field splitting is largest in octahedral coordination, followed by cubic coordination and then dodecahedral coordination.

**References**

1. Y. Xiao, W. Xiao, D. Wu, L. Guan, M. Luo, L.-D. Sun. An extra‐broadband VIS‐NIR emitting phosphor toward multifunctional LED Applications. Advanced Functional Materials. 2022, 32, 2109618.
2. N. Yamashita, S. Maekawa, K. Nakamur, Absorption lines of manganous fluosilicate hexahydrte. Japanese Journal of Applied Physics 1990, 29, 1729−1732.
3. C. R. Ronda, T. Amrein, Evidence for exchange-induced luminescence in Zn_2_SiO_4_:Mn. Journal of Luminescence. 1996, 69, 245−248.
4. A. P. Vink, M. A. de Bruin, S. Roke, P. S. Peijzel, A. Meijerink. Luminescence of exchange coupled pairs of transition metal ions. Journal of The Electrochemical Society 2001, 148, E313−E320.
5. Z. G. Xia, Y. Y. Zhang, M. S. Molokeev, V. V. Atuchin. Structural and luminescence properties of yellow-Emitting NaScSi_2_O_6_:Eu^2+^ phosphors: Eu^2+^ site preference analysis and generation of red emission by codoping Mn^2+^ for white-light-emitting diode applications. Journal of Physical Chemistry C 2013, 117, 20847−20854.
6. M. L. Deng, M. M. Shang, Y. X. Sun, Y. N. Wang, X. L. Xing. Thermally stable red-emitting Ca_18_K_3_Sc(PO_4_)_14_:Mn^2+^ phosphor and enhanced luminescence by energy transfer between Ce^3+^–Eu^2+^–Mn^2+^. Inorganic Chemistry, 2024, 63, 3901–3912.
7. W. J. Gong, J. B. Luo, W. Y. Zhou, J. Q. Fan, Z. S. Sun, S. X. Zeng, H. W. Pan, Z. P. Zhu, X. X. Yang, Z. Q. Yu, X. G. Zhang. Thermal-stable blue-red dual-emitting Na_2_Mg_2_Si_6_O_15_:Eu^2+^, Mn^2+^ phosphor for plant growth lighting. Journal of Luminescence, 2021, 239, 118372.
8. E. Song, X. Jiang, Y. Zhou, Z. Lin, S. Ye, Z. Xia, Q. Zhang. Heavy Mn^2+^ doped MgAl_2_O_4_ phosphor for high-efficient near-infrared light-emitting diode and the night-vision application. Advanced Optical Materials, 2019, 7, 1901105.
9. X. Li, T. Liu, K. Zhang, Z. Hu, H. An, S. Deng, Y. Kong, B. Wang. Highly efficient Mn–Mn dimer activated phosphors for high-power near-infrared LED application. Journal of Materials Chemistry C, 2023, 11, 712–721.
10. C. Y. Zhan, H. M. Zhu, S. S. Liang, W. D. Nie, Z. H. Wang, M. C. Hong. Mn^2+^–Mn^2+^ dimers induced robust light absorption in heavy Mn^2+^ doped ZnAl_2_O_4_ near-infrared phosphor with an excellent photoluminescence quantum yield and thermal stability. Advanced Optical Materials, 2024, 12, 2400574.
11. A. J. van Bunningen, S. T. Keizer, A. Meijerink Understanding enormous redshifts in highly concentrated Mn^2+^ phosphors. Journal of Materials Chemistry C, 2023, 11, 8961–8970.
12. A. J. van Bunningen, A. D. Sontakke, R. van der Vliet, V. G. Spit, A. Meijerink. Luminescence temperature quenching in Mn^2+^ phosphors. Advanced Optical Materials, 2023, 11, 2202794.
13. D. L. Griscom, R. E. Griscom, Paramagnetic resonance of Mn^2+^ in glasses and compounds of the lithium borate system. The Journal of Chemical Physics, 1967, 47, 2711.
14. V. Singh, R. P. S. Chakradhar, J.L. Rao, H.-Y. Kwak. Green luminescence and EPR studies on Mn-activated yttrium aluminum garnet phosphor. Applied. Physics. B 2010, 98, 407−415.
15. I. N. Prakash, B. Babu, Ch. Venkata Reddy, P. Narayana Murty, Y. P. Reddy, P. Sambasiva Rao, R.V. S. S. N. Ravikumar, Spectroscopic studies on Fe^3+^ and Mn^2+^ doped SrB_4_O_7_ glasses. Physica B 2011, 406, 3295–3298.
16. K. G. Santosh, R. M. Kadam, R. Gupta, S. Manjulata, V. Natarajan. Evidence for the stabilization of manganese ion as Mn (II) and Mn (IV) in a-Zn_2_P_2_O_7_: Probed by EPR, luminescence and electrochemical studies. Materials Chemistry and Physics, 2014, 145, 162–167.
17. W. Tang, T. Fu, K. Deng, M. Wu, Preparation of Ca_4_Mg_5_(PO_4_)_6_:Eu^2+^, Mn^2+^ phosphor and its photoluminescence properties. Ceramics International, 2013, 39, 6363–6367.
18. H. Jiao, F. Liao, S. Tian, X. Jing, Luminescent properties of Eu^3+^ and Tb^3+^ activated Zn_3_Ta_2_O_8_, Journal of the Electrochemical Society 2003, 150(9), H220–H224.
19. P. Paulose, G. Jose, V. Thomas, N. Unnikrishnan, M. Warrier. Sensitized fluorescence of Ce^3+^/Mn^2+^ system in phosphate glass. Journal of Physics and Chemistry of Solids, 2003, 64, 841–846.
20. D. L. Dexter. A Theory of sensitized luminescence in solids. The Journal of Chemical Physics, 1953, 21(5), 836–850.
21. X. Zhang, L. Huang, F. Pan, M. Wu, J. Wang, Y. Chen, Q. Su. Highly thermally stable single-component white-emitting silicate glass for organic-resin-free white-light-emitting Diodes. ACS Applied Materials & Interfaces 2014, 6, 2709−2717
22. P. Dorenbos. Electronic structure and optical properties of the lanthanide activated RE_3_(Al_1-_ *_x_*Ga*_x_*)_5_O_12_ (RE = Gd, Y, Lu) garnet compounds. Journal of Luminescence, 2013, 134, 310–318.
23. Z. G. Xia, A. Meijerink. Ce^3+^-Doped garnet phosphors: composition modification, luminescence properties and applications. Chemical Society Reviews, 2017, 46, 275–299.
